# EGR3-HDAC6-IL-27 Axis Mediates Allergic Inflammation and Is Necessary for Tumorigenic Potential of Cancer Cells Enhanced by Allergic Inflammation-Promoted Cellular Interactions

**DOI:** 10.3389/fimmu.2021.680441

**Published:** 2021-06-21

**Authors:** Yoojung Kwon, Misun Kim, Youngmi Kim, Myeong Seon Jeong, Hyun Suk Jung, Dooil Jeoung

**Affiliations:** ^1^ Department of Biochemistry, Kangwon National University, Chuncheon, South Korea; ^2^ Institute of New Frontier Research, College of Medicine, Hallym University, Chuncheon, South Korea; ^3^ Chuncheon Center, Korea Basic Science Institute, Chuncheon, South Korea

**Keywords:** allergic inflammation, cellular interactions, EGR3, HDAC6, IL-27, MiR-182-5p

## Abstract

The objective of this study was to investigate mechanisms of allergic inflammation both *in vitro* and *in vivo* in details. For this, RNA sequencing was performed. Early growth response 3 gene (Egr3) was one of the most highly upregulated genes in rat basophilic leukemia (RBL2H3) cells stimulated by antigen. The role of Egr3 in allergic inflammation has not been studied extensively. Egr3 was necessary for passive cutaneous anaphylaxis (PCA) and passive systemic anaphylaxis (PSA). Egr3 promoter sequences contained potential binding site for NF-κB p65. NF-κB p65 directly regulated Egr3 expression and mediated allergic inflammation *in vitro*. Histone deacetylases (HDACs) is known to be involved in allergic airway inflammation. HDAC6 promoter sequences contained potential binding site for EGR3. EGR3 showed binding to promoter sequences of HDAC6. EGR3 was necessary for increased expression of histone deacetylase 6 (HDAC6) in antigen-stimulated RBL2H3 cells. HDAC6 mediated allergic inflammation *in vitro* and PSA. TargetScan analysis predicted that miR-182-5p was a negative regulator of EGR3. Luciferase activity assay confirmed that miR-182-5p was a direct regulator of EGR3. MiR-182-5p mimic inhibited allergic inflammation both *in vitro* and *in vivo*. Cytokine array showed that HDAC6 was necessary for increased interleukin-27 (IL-27) expression in BALB/C mouse model of PSA. Antigen stimulation did not affect expression of EBI3, another subunit of IL-27 in RBL2H3 cells or BALB/C mouse model of PCA or PSA. IL-27 receptor alpha was shown to be able to bind to HDAC6. IL-27 p28 mediated allergic inflammation *in vitro*, PCA, and PSA. Mouse recombinant IL-27 protein promoted features of allergic inflammation in an antigen-independent manner. HDAC6 was necessary for tumorigenic and metastatic potential enhanced by PSA. PSA enhanced the metastatic potential of mouse melanoma B16F1 cells in an IL-27-dependent manner. Experiments employing culture medium and mouse recombinant IL-27 protein showed that IL-27 mediated and promoted cellular interactions involving B16F1 cells, lung macrophages, and mast cells during allergic inflammation. IL-27 was present in exosomes of antigen-stimulated RBL2H3 cells. Exosomes from antigen-stimulated RBL2H3 cells enhanced invasion of B16F1 melanoma cells in an IL-27-dependemt manner. These results present evidence that EGR3-HDAC6-IL-27 axis can regulate allergic inflammation by mediating cellular interactions.

## Introduction

FcϵRI signaling contributes to the pathogenesis of systemic anaphylaxis, passive cutaneous anaphylaxis (PCA), passive systemic anaphylaxis (PSA) (1–3), and atopic dermatitis (4). Systemic anaphylaxis is accompanied by hypotension, decreased body temperature, and increased β-hexosaminidase activity (5). Cellular interactions involving mast cells, macrophages, and many other immune cells contribute to the pathogenesis of anaphylaxis (6). Exosomes mediate these cellular interactions (7).

Early growth response gene 3 (Egr3) is necessary for the upregulation of both IL-6 and IL-8 (8). Both IL-6 and IL-8 serve as direct targets of Egr3 (8). Both IL-6 and IL-8 play important roles in airway smooth muscle cell inflammation (9). Thus, Egr 3 may allergic inflammation such as anaphylaxis.

Epigenetic modifications contribute to the pathogenesis of allergic diseases. FcϵRI-HDAC3-MCP1 axis mediates allergic inflammations both *in vitro* and *in vivo* (7). DNA methyltransferase I (DNMT1) can suppress allergic skin inflammation (10). A low level of DNMT1 contributes to the pathogenesis of asthma (11). Histone deacetylases (HDACs) play critical roles in house dust mite (HDM)-induced allergic airway inflammations (12). T cell-specific loss of HDAC1 increases inflammatory response in a mouse model of asthma (13). Decreased expression of HDAC2 is responsible for allergic airway inflammation (14) and PCA (15). HDAC3 can bind to FcϵRI and mediate PCA and PSA (15, 16). Tubastatin A can suppress airway inflammation by inhibiting histone deacetylase 6 (HDAC6), thereby decreasing levels of Th2 cytokines (17).

Activation of TLR4-NF-κB contributes to the pathogenesis of allergic contact dermatitis (18). TLR2/TLR4/MyD88-signaling pathway mediates allergic asthma (19). HDAC6 is necessary for the activation of TLR4-MAPK/NF-κB signaling in LPS-induced inflammation (20). These reports imply that TLR-HDAC6-MAPK- NF-κB signaling might regulate allergic inflammation such as anaphylaxis.

IL-27 enhances proinflammatory responses by increasing TLR4 expression in a NF-kB-dependent manner (21). IL-27 mediates the increase of IL-1 beta by LPS in human monocytes (22).

MicroRNAs (miRNAs) are non-coding small RNAs that contribute to the pathogenesis of allergic diseases. MiR-20a-5p targets HDAC4 and suppresses allergic inflammation in human mast cells (HMC-1) (23). MiR-34a can mediate allergic asthma by increasing forkhead box P3 (FOXP3) expression (24). MiR-142-3p affects the balance between proliferation and differentiation of airway smooth muscle cells in asthma by regulating WNT signaling (25). MiR-133b can inhibit allergic rhinitis by targeting NOD-like receptor pyrin domain-containing protein 3 (Nlrp3) inflammasome-meditated inflammation (26). MiR-155 can mediate allergic airway inflammation by regulating IL-33 signaling (27). These reports suggest that miRNAs play important roles in anaphylaxis.

Global-level identification of antigen-regulated genes is critical for understanding the mechanism that contributes to the pathogenesis of anaphylaxis. In the present study, RNA sequencing analysis revealed that Egr3 was one of the most highly upregulated genes in rat basophilic leukemia cells (RBL2H3) stimulated with antigen. We found that EGR3 regulated the expression of HDAC6 which was necessary for increased expression of IL-27 during allergic inflammation. MiR-182-5p targeted Egr3 and negatively regulated anaphylaxis. Cellular interactions contributed to the tumorigenic and metastatic potential of cancer cells enhanced by PSA. IL-27 was present in exosomes and mediated cellular interactions during allergic inflammation. Our results presented novel roles of EGR3-HDAC6-IL-27 axis in allergic inflammation.

## Materials and Methods

### Materials

We purchased oligonucleotides from Bioneer Company (Daejeon Korea). We purchased DNP-HSA (2, 4-dinitrophenyl-human serum albumin) and DNP-specific IgE antibody from Sigma. Anti-mouse and anti-rabbit IgG-horseradish peroxidase-conjugated antibody were purchased from Pierce. We purchased other antibodies from Cell Signaling Co. (Beverly, MA). We purchased *in vitro* transfection agents (Lipofectamine and PlusTM reagent) from Invitrogen. We purchased mouse recombinant IL-27 protein from R&D systems.

### Cell Culture

We purchased rat basophilic leukemia (RBL2H3) cells and mouse melanoma B16F1 cells from the Korea Cell Line Bank. We isolated lung mast cells, lung macrophages, and bone marrow-derived mast cells, according to the standard procedures (7).

### Mice

We purchased female BALB/C mice Nara Biotech (Seoul, Korea). All animal experiments were approved by the Institutional Animal Care and Use Committee (IACUC) of Kangwon National University.

### RNA Sequencing and Analysis

TRIzol^®^ RNA Isolation Reagents (Life technologies) were employed for extraction of total RNA. Total RNA was then processed for preparing mRNA sequencing library using the Illumina TruSeq Stranded mRNA Sample Preparation kit (Illumina). All libraries were quantified by qPCR (CFX96, Biorad) and sequenced on the NextSeq500 sequencers (Illumina) with a paired-end 75bp plus single 8bp index read run. To quantify the mapped reads on the reference genome into the gene expression values, Cufflinks (28) with the strand-specific library option and other default options was used. The differentially expressed genes were analyzed by Cuffdiff software (29) with the strand-specific library option. To compare the expression profiles among the samples, the normalized expression values of the selected a few hundred of the differentially expressed genes were unsupervised clustered by in-house R scripts.

### Data Availability

The RNAseq data sets analyzed for this study can be found at the NCBI’s Sequence Read Archive (https://www.ncbi.nlm.nih.gov/sra) (PRJNA606652).

### Quantitative Real Time PCR

Total miRNA isolated by miRNeasy Mini Kit (QIAGEN) was extended and synthesized from miRNA to cDNA according to the standard procedures (Sigma-Aldrich). Determination of miR-182-5p expression level was based on the threshold (*Ct*), and relative expression level was determined as 2^−(Ct^ *^ofmiR^* ^−182−5^ *^p^* ^)−(^ *^CtofU^* ^6)^ after normalization with reference to expression of U6 small nuclear RNA. For quantitative real-time PCR, SYBR PCR Master Mix (Applied Biosystems) was used in a CFX96 Real Time System thermocycler (Bio-Rad). The primer sequences for qRT-PCR are provided in **Supplementary Tables**.

### Constructs

Gene segment encompassing 3′-UTR of rat Egr3 (758 bp) was PCR-amplified and cloned into the pGL3 luciferase plasmid. QuikChange site-directed mutagenesis kit (Stratagene) was used to make mutant pGL3–3′-UTR-Egr3 construct. Gene segment encompassing full-length rat *HDAC6* promoter (736 bp) or rat *Egr3* promoter (943 bp) was PCR-amplified and cloned into the pGL2 basic luciferase plasmid. Promoter deletion constructs were also made by PCR amplification and cloning into the pGL2 basic luciferase plasmid. Luciferase activity was determined as described (7).

### Transfections

The negative control siRNA was purchased from Bioneer Company (cat.SN-1002). For *in vivo* transfections, *in vivo*-jetPEI^®^ (Polyplus, cat.201-10G) was used. The sequences of miR-mimic and siRNAs are listed in **Supplementary Tables**.

### Cytokine Array

Cytokine array analysis (Proteom ProfilerTM Mouse Cytokine Array Kit) was performed as described (R&D system).

### MiRNA Target Analysis

TargetScan program identified targets of miR-182-5p.

### Chromatin Immunoprecipitation Assay

The RBL2H3 cells were cross-linked and ChIP DNA was isolated. EGR3-specific antibody, NF-kB p65-specific antibody and IgG control were used for ChIP assay. PCR was done with specific primers of the HDAC6 promoter-1 (5′-TGGGCGGGCAAATGAAAAAG-3′ (sense) and 5′-GCCTACCGT TTAACCAGGCT-3′(antisense)), HDAC6 promoter-2(5′- GGATTC TGATCGAAAGGGGCA-3′ (sense) and 5′-TCCACTTCCCACATCCTTTCAT-3′ (antisense)), and HDAC6 promoter-3 (5′-GGGT AGGGCAGGCCTAAGAA-3′ (sense) and 5′- CTAGATCGCA GCCTTCACCG-3′ (antisense)) sequences were used to determine the binding of EGR3. Egr3 promoter-1 (5′- GGGTTGA AGCGGTCATCTCC -3′ (sense) and 5′- ACCGCTCGCCGTTCTTTATG -3′ (antisense)), Egr3 promoter-2 (5′- CATAAAG AACGGCGAGCGGT -3′ (sense) and 5′- ACCTCCTCTGCTGCTGCT -3′ (antisense)), and Egr3 promoter-3 (5′- GCGCGTGTCTGTGAGATCA -3′ (sense) and 5′- CTTCCAGGCTAGCGGCAT -3′ (antisense)) were used to determine the binding of NF-kB p65.

### β-hexosaminidase Activity Assays

The β-hexosaminidase activity was performed according to the standard procedures (7). The detailed procedures are in **Supplementary Methods**.

### Immunoblot and Immunoprecipitation

Immunoblot and immunoprecipitation were performed according to the standard procedures with some modifications. The detailed procedures are in **Supplementary Methods**. The lists of antibodies are in **Supplementary Table**.

### Immunofluorescence Staining

Cells were subjected to fixing (4% paraformaldehyde (v/v) and then permeabilization (0.4% Triton X-100) and incubated with anti-IL-27 receptor (BIO-RAD), anti-NF-kB p65 (Cell signaling) or anti-HDAC6 antibody (ABclonal) for 2 h. For detection of IL-27 receptor and NF- kB, anti-rabbit Alexa Fluor 488 secondary antibody (Molecular probes) was added to cells and incubated for 1 h. For detection of HDAC6, anti-goat Alexa Fluor 546 (Molecular probes) was employed. For fluorescence imaging, confocal laser scanning microscope and software (Fluoview version 2.0) with a X 60 objective (Olympus FV300, Tokyo, Japan) were employed. The lists of antibodies are described in **Supplementary Table**.

### Immunohistochemical Staining

Sections of the paraffin-embedded tissue blocks (4–6 μm-thick) were mounted on positively charged glass slides, and dried in an oven at 56°C for 30 min. The sections were deparaffinized and then rehydrated, and hydrogen peroxide was added to suppress endogenous peroxidase. After treatment with bovine serum albumin (BSA) to block nonspecific binding, the sections were then incubated with primary antibody overnight at 4°C. After washing, biotinylated secondary antibody was added for 1 h. Diaminobenzidine (Vector Laboratories, Inc.) was employed for color development. Mayer’s hematoxylin was used for counterstaining of sections. The lists of antibodies are described in **Supplementary Table**.

### Passive Cutaneous Anaphylaxis

The induction of passive cutaneous anaphylaxis (PCA) in BALB/C mice was performed as described (7). To determine the effect of miR-182-5p on the PCA, BALB/C mice were giving an intradermal injection with DNP-IgE (0.5 μg/kg) and also intravenously injected with control mimic (3 μg/kg) or miR-182-5p mimic (3 μg/kg). The next day, BALB/C mice were intravenously injected with PBS or DNP-HSA (250 μg/kg) along with 2% (v/v) Evans blue solution. To determine the effect of Egr3 on the PCA, BALB/C mice were given an intradermal injection of DNP-IgE (0.5 μg/kg) and intravenously injected with negative control siRNA (3 μg/kg) or Egr3 siRNA (3 μg/kg). The next day, BALB/C mice were intravenously injected with PBS or DNP-HSA (250 μg/kg) along with 2% (v/v) Evans blue solution.

### Passive Systemic Anaphylaxis

The induction of passive systemic anaphylaxis (PSA) in BALB/C mice was performed as described (7). To examine the effect of HDAC6 on PSA, BALB/C mice were intravenously injected with DNP-specific IgE (0.5 μg/kg) along with control siRNA (3 μg/kg) or HDAC6 siRNA (3 μg/kg). Twenty-four hours later, mice were intravenously injected with DNP-HSA (250 μg/kg).

### Effect of Passive Systemic Anaphylaxis on Tumorigenic and Metastatic Potential

PSA was induced as described. To determine the effect of HDAC6 on tumorigenic potential and metastatic potential enhanced by PSA, BALB/C mice were intravenously injected with control siRNA (3 μg/kg) or HDAC6 siRNA (3 μg/kg) on the indicated days as described in **Figure 7**. To determine the effect of IL-27 on metastatic potential enhanced by PSA, BALB/C mice were intravenously injected with isotype-matched IgG (20 μg/kg) or nIL-27 antibody (20 μg/kg) on the indicated days as described in **Figure 8**.

### Isolation of Exosomes

Exosomes were purified using Total Exosome Isolation Reagent (ThermoFisher, USA) and observed under a Tecnai T10 transmission electron microscope (FEI, USA).

### Shuttling of Exosomes Between Cells

PKH67 Fluorescent Cell Linker kits (Sigma-Aldrich, St. Louis, MO) were employed for labeling of exosomes. To examine the uptake of exosomes, PKH67-labeled or PKH67-unlabeled exosomes were added into unstimulated RBL2H3 cells on coverslip (2 × 10^4^ cells) for 24 h, followed by washing and fixing with paraformaldehyde solution (4% v/v) for 15 min. The uptake of exosomes was visualized under a confocal laser scanning microscope LX70 FV300 05-LPG-193 (Olympus).

### The Presence of IL-27 in the Exosomes

The presence of IL-27 in the exosomes was determined according to the standard procedures (7). Collected extracellular vesicles were treated with fixing solution (7) for 1 h at 4°C and then osmium tetroxide 2%) for 30 min at 4°C. They were dehydrated with a graded series of ethanol followed by treatment with graded propylene oxide series, and embedded into epoxy resin (PELCO, USA). Ultrathin sections (~80 nm) obtained with Ultracut UCT (Leica, Germany) were mounted on copper grids and stained with 1% uranyl acetate and lead citrate (10 min). For immune-gold labeling electron microscopy, ultrathin sections on the grids were treated with 0.02 M glycine for 10 min. Sections were washed for 1 h in PBS, and incubation with the primary rabbit or mouse antibody (Anti-IL-27 p28 or/and Anti-TSG101 antibody at 1:20 dilution) for overnight at 4°C was followed. The grids were washed with 0.1% BSA in PBS, incubation with anti- Rabbit IgG conjugated to 10 nm or anti-mouse IgG conjugated to 25 nm (AURION, Holland) was followed. The grids were examined using a Tecnai T10 transmission electron microscope (FEI, USA) and JEOL-2100F transmission electron microscope (JEOL, USA).

### Monitoring of Rectal Temperature

Rectal temperatures associated with passive systemic anaphylaxis were monitored by using a digital thermometer.

### Statistical Analysis

Data were analyzed and graphed using GraphPad Prism statistics program (GraphPad Prism software). Results are presented as means ± S.E. Student’s t tests were performed for comparisons between two groups. One-way ANOVA was carried out for comparisons among three or more groups and was followed by Tukey’s *post hoc* test. Values were considered significant at p value less than 0.05.

## Results

### Antigen Stimulation Increased EGR3 Which Mediates Allergic Inflammation *In Vitro*

**Figure 1:**
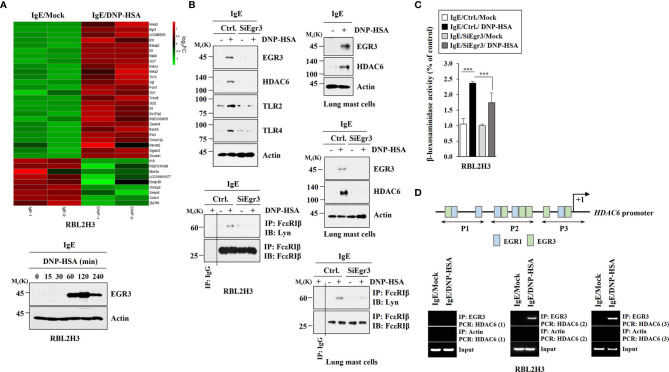
Antigen stimulation increases EGR3 that mediates allergic inflammation *in vitro* and EGR3 binds to the promoter sequences of HDAC6. **(A)** The DNP-specific IgE-sensitized RBL2H3 cells were treated without or with DNP-HSA (100 ng/ml) for 1 h. Total RNAs were subjected to RNA sequencing analysis (upper panel). RNA sequencing analysis represents replicates within one experiment. Cell lysates prepared at each time point were subjected to immunoblot (lower). Representative blots of three independent experiments are shown. **(B)** The indicated siRNA (each at 10 nM) was transfected into RBL2H3 cells (left) or lung mast cells (right). At 24 hours after transfection, cells were sensitized with IgE for 24 h, followed by stimulation with DNP-HSA for 1 h. Ctrl. denotes negative control siRNA. The IgE-sensitized lung mast cells were stimulated with DNP-HSA for 1 h (upper, right). Immunoblot and immunoprecipitation were performed. Immunoprecipitation using isotype-matched IgG antibody (2 μg/ml) was also performed. Representative blots of three independent experiments are shown. **(C)** The β-hexosaminidase activity assays were performed in RBL2H3 cells. ***p<0.001. Average values of three independent experiments are shown. **(D)** ChIP assays were performed in RBL2H3 cells treated without or with DNP-HSA (100 ng/ml) for 1 h. P1, P2, and P3 denote primer-binding sites. Anti-actin antibody served as a control antibody.

We wanted to investigate the mechanism of allergic inflammation. To identify genes that could regulate allergic inflammation, RNA sequencing analysis was performed using rat basophilic leukemia (RBL2H3) cells (**Figure 1A**). RNA sequencing revealed that early growth response 3 (Egr3) was one of the most highly upregulated genes in RBL2H3 cells stimulated with antigen. Antigen stimulation increased the expression of EGR3 in RBL2H3 cells in a time-dependent manner (**Figure 1A**). RNA interference decreased the expression of Egr3 which inhibited expression levels of HDAC6, TLR2, and TLR4 in RBL2H3 cells and lung mast cells increased by antigen stimulation (**Figure 1B**). TLR2 (30) and TLR4 (31) mediate allergic airway inflammation and atopic dermatitis, respectively. Decreased expression of Egr3 by RNA interference inhibited interaction between FcϵRI and Lyn in RBL2H3 cells and lung mast cells induced by antigen stimulation (**Figure 1B**). Decreased expression of Egr3 by RNA interference inhibited the increase of β-hexosaminidase activity in RBL2H3 cells induced by antigen stimulation (**Figure 1C**). EGR3 and HDAC6 serve as targets of estrogen receptor α (32). HDAC6 promoter sequences contain potential binding site for Egr3 (**Figure 1D**). EGR3 was shown to be able to bind to promoter sequences of HDAC6 in antigen-stimulated RBL2H3 cells (**Figure 1D**). Antigen stimulation increased luciferase activity associated with full-length *HDAC6* promoter (pGL2-HDAC6) in RBL2H3 cells (**Figure S1**). Deletion of site 1 (pGL2-Del628) or sites 1 and 2 (pGL2-Del336) of *HDAC6* promoter sequences did not prevent antigen from increasing luciferase activity associated with *HDAC6* promoter in RBL2H3 cells (**Figure S1**). However, deletions of both site 2 and site 3 (pGL2-Del849) of HDAC6 promoter sequences prevented antigen from increasing luciferase activity associated with *HDAC6* promoter in RBL2H3 cells (**Figure S1**). These results indicate that EGR3 binds to site 2 (P2) and site 3 (P3) of *HDAC6* promoter sequences to increase HDAC6 expression in response to antigen. Therefore, Egr3 might mediate allergic inflammation *in vitro* by increasing HDAC6 expression.

### NF-kB p65 Directly Regulates the Expression of Egr3 and Mediates Allergic Inflammation *In Vitro*

**Figure 2:**
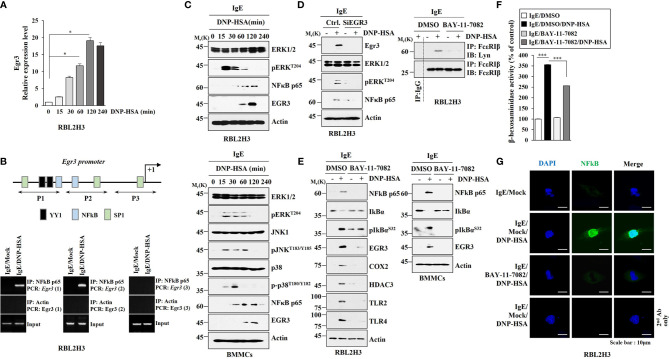
NF-κB directly regulates EGR3 expression and mediates allergic inflammation *in vitro*. **(A)** Cell lysates prepared at each time point were subjected to qRT-PCR. *p<0.05. Average values of three independent experiments are shown. **(B)** Shows promoter sequences of Egr3 with potential binding sites for the indicated transcriptional factors. ChIP assays were then performed in RBL2H3 cells treated without or with DNP-HSA for 1h. P1, P2, and P3 denote primer-binding sites. Anti-actin antibody served as a control antibody. **(C)** The IgE-sensitized RBL2H3 cells (upper) or BMMCs (lower) were treated with DNP-HSA for various time intervals. Representative blots of three independent experiments are shown. **(D)** RBL2H3 cells were transfected with the indicated siRNA (each at 10 nM). The next day, cells were then sensitized with IgE for 24 h, and then stimulated by DNP-HSA for 1 h. Representative blots of three independent experiments are shown. **(E)** The IgE-sensitized RBL2H3 cells (left) or BMMCs (right) were treated without or with BAY-11-7082 (10 μM) for 1 h. Cells were then stimulated without or with DNP-HSA for 1h, followed by immunoblot and immunoprecipitation (right upper). Representative blots of three independent experiments are shown. **(F)** The β-hexosaminidase activity assays were performed in RBL2H3 cells. ***p<0.001. Average values of three independent experiments are shown. **(G)** Immunofluorescence staining was performed in RBL2H3 cells.

Since antigen increased EGR3 expression in RBL2H3 cells and lung mast cells, we investigated the mechanism of expression regulation of Egr3. Antigen increased Egr3 expression in RBL2H3 cells at the transcriptional level in a time-dependent manner (**Figure 2A**). This suggests that transcriptional factors might regulate EGR3 expression. Egr3 promoter sequences showed the presence of potential binding sites for YY1, NF-κB, and SP1 (**Figure 2B**). We hypothesized that these transcription factor(s) might increase Egr3 expression. NF-κB p65 was shown to be able to bind to promoter sequences of Egr3 in antigen-stimulated RBL2H3 cells (**Figure 2B**). Antigen increased expression levels of pERK^T204^, NF-κB p65, pJNK^T183/Y185^, pp38MAPK^T180/Y182^, and EGR3 in bone marrow-derived mast cells (BMMCs) in a time-dependent manner (**Figure 2C**). Decreased expression of Egr3 by RNA interference decreases of expression levels of pERK^T204^ and NF-κB p65 in RBL2H3 cells induced by antigen stimulation (**Figure 2D**). This suggests that Egr3 and NF-κB p65 might form a positive feedback loop to regulate allergic inflammation. Egr3 might regulate expressions of NF-kB p65 regulators. It is necessary to identify EGR3-regulated genes to understand the mechanism of increase of NF-κB p65 by allergic inflammation. BAY-11-7082, an NF-κB p65 inhibitor, suppressed the interaction between FcϵRI and Lyn in RBL2H3 cells (**Figure 2D**, right). BAY-11-7082 exerted negative effects on increases of expression levels of NF-κB p65, pIkBα^S32^, EGR3, COX2, HDAC3, TLR2, and TLR4 induced by antigen in RBL2H3 cells (**Figure 2E**). BAY-11-7082 inhibited decrease of IκBα expression induced by antigen stimulation (**Figure 2E**). BAY-11-7082 also prevented the antigen from increasing expression levels of NF-κB p65, EGR3, and pIkBα^S32^ in bone marrow-derived mast cells (BMMCs) (**Figure 2E**). BAY-11-7082 inhibited the increase of β-hexosaminidase activity in RBL2H3 cells induced by antigen stimulation (**Figure 2F**). Immunofluorescence staining also showed an increased expression of NF-κB p65 expression in RBL2H3 cells induced by antigen (**Figure 2G**). Antigen stimulation increased luciferase activity associated with full-length *EGR3* promoter (pGL2-Egr3) in RBL2H3 cells (**Figure S2**). Deletion of site 1 (pGL2-Del677) did not prevent antigen from increasing luciferase activity associated with *EGR3* promoter in RBL2H3 cells (**Figure S2**). However, deletions of both site 1 and site 2 of *EGR3* promoter (pGL2-Del326) prevented antigen from increasing luciferase activity associated with *EGR3* promoter in RBL2H3 cells (**Figure S2**). These results suggest that NF-kB p65 binds to site 1 (P1) and site 2 (P2) of *EGR3* promoter sequences to increase EGR3 expression in response to antigen in RBL2H3 cells. Thus, NF-κB p65 can mediate allergic inflammation by directly increasing EGR3 expression.

### Egr3 Mediates PCA and PSA

**Figure 3:**
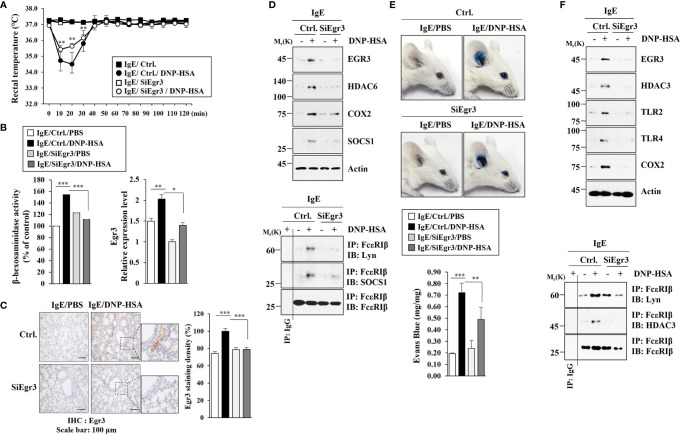
Egr3 is necessary for anaphylaxis. **(A)** BALB/C mice were intravenously injected with the indicated siRNA (each at 3 μg/kg). The next day, BALB/C mice were given an intravenous injection of IgE (0.5 μg/kg). The following day, BALB/C mice were intravenously injected with DNP-HSA (250 μg/kg), and rectal temperatures were measured. Each experimental group comprised five mice. The means ± S.E. of three independent experiments were shown. **p<0.01, compared with IgE/Ctrl./DNP-HSA. **(B)** Lung tissue lysates were subjected to β-hexosaminidase activity assays and qRT-PCR. *, p<0.05; **p<0.01; ***p<0.001. The data are expressed as mean ± SE of results from four mice of each experimental group. **(C)** Immunohistochemical staining employing lung tissues was performed. Representative images of the staining are shown (n=3). Quantification was performed by calculating the percentage of the staining intensities using Image J (NIH). ***p<0.001. **(D)** Immunoblot and immunoprecipitation employing lung tissue lysates were performed (n=4). Representative blots of three independent experiments are shown. **(E)** BALB/C mice were given an intradermal injection of IgE (0.5 μg/kg) and an intravenous injection of the indicated siRNA (each at 3 μg/kg). The next day, BALB/C mice were intravenously injected with PBS or DNP-HSA (250 μg/kg) along with 2% (v/v) Evans blue solution. Each experimental group comprised four BALB/C mice. **p<0.01; ***p<0.001. **(F)** Immunoblot and immunoprecipitation employing ear tissue lysates were performed (n=4). Representative blots of three independent experiments are shown.

Since Eg3 expression was increased during allergic inflammation *in vitro*, we examined the role of Egr3 in allergic inflammation *in vivo*. Egr3 was necessary for the decrease of rectal temperature by passive systemic anaphylaxis (PSA) in BALB/C mice (**Figure 3A**). The decrease of Egr3 expression by RNA interference inhibited the increase of β-hexosaminidase activity in BALB/C mice induced by PSA (**Figure 3B**). PSA increased Egr3 expression at the transcriptional level in BALB/C mice (**Figure 3B**). Immunohistochemical staining employing lung tissues showed that PSA increased EGR3 expression (**Figure 3C**). The decreased expression of Egr3 by RNA interference inhibited increases of the expression levels of HDAC6, COX2, and SOCS1 in lung tissue lysates induced by PSA based on immunoblot (**Figure 3D**). The decreased expression of Egr3 by RNA interference also inhibited interactions of FcϵRI with Lyn and SOCS1 in lung tissue lysates induced by PSA based on immunoprecipitation (**Figure 3D**). Egr3 mediated passive cutaneous anaphylaxis (PCA) in BALB/C mice (**Figure 3E**). Immunoblot of ear tissue lysates showed that the decreased expression of Egr3 by RNA interference inhibited increases of expression levels of HDAC3, TLR2, TLR4, COX2, and SOCS1 induced by PCA (**Figure 3F**). Immunoprecipitation of ear tissue lysates showed that decreased expression of Egr3 by RNA interference inhibited interactions of FcϵRI with Lyn and HDAC3 induced by PCA (**Figure 3F**). Thus, Egr3 can mediate anaphylaxis. Further studies are needed to investigate the mechanism of Egr3-mediated anaphylaxis.

### HDAC6 Mediates Passive Systemic Anaphylaxis

**Figure 4:**
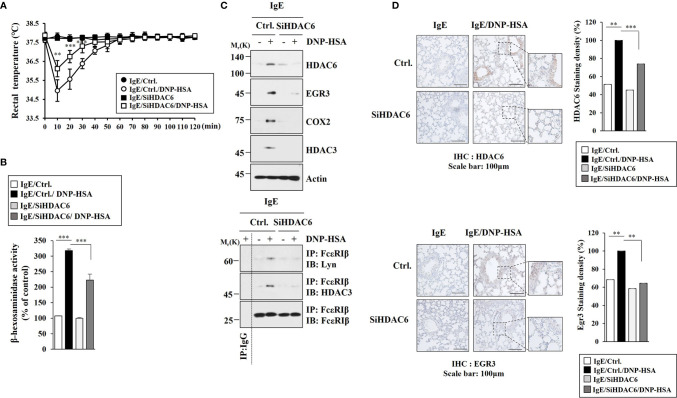
HDAC6 mediates passive systemic anaphylaxis. **(A)** BALB/C mice were intravenously injected with the indicated siRNA (each at 3 μg/kg). Each experimental group comprised five mice. The means ± S.E. of three independent experiments are depicted. **p<0.01, compared with IgE/Ctrl./DNP-HSA; ***p<0.001, compared with IgE/Ctrl./DNP-HSA. **(B)** The β-hexosaminidase activity assays were performed. ***p<0.001. The data are expressed as mean ± SE of results from four mice of each experimental group. Average values of three independent experiments are shown. **(C)** Immunoblot and immunoprecipitation employing lung tissue lysates were performed (n=4). Representative blots of three independent experiments are shown. **(D)** Immunohistochemical staining employing lung tissue was performed. Representative images of the staining are shown (n=3). Quantification was performed using Image J (NIH). **p<0.01; ***p<0.001.

EGR3 could bind to promoter sequences of HDAC6 (**Figure 1D**). Anaphylaxis increased the expression of HDAC6 in an Egr3-dependent manner (**Figure 3D**). We therefore examined the effect of HDAC6 on anaphylaxis. Decreased expression of HDAC6 by RNA interference inhibited decrease of rectal temperature (**Figure 4A**) and increase of β-hexosaminidase activity in BALB/C mice induced by PSA (**Figure 4B**). Decreased expression of HDAC6 by RNA interference inhibited the effect of PSA on increases of expression levels of EGR3, TLR2, TLR4, COX2, and HDAC3 (**Figure 4C**) and interactions of FcϵRI with Lyn and HDAC3 in BALB/C mice induced by PSA (**Figure 4C**). Immunohistochemical staining employing lung tissues of BALB/C mice showed that HDAC6 was necessary for the increased EGR3 expression by PSA (**Figure 4D**). Thus, HDAC6 can mediate anaphylaxis by regulating FcϵRI signaling.

### MiR-182-5p Targets Egr3 and Negatively Regulates Allergic Inflammation *In Vitro*

Since miRNAs play important roles in anaphylaxis (2, 7), we hypothesized that miRNAs that regulate Egr3 could play critical roles in anaphylaxis. TargetScan analysis predicted that miR-182-5p was a negative regulator of Egr3. MiR-182-5p was shown to be a direct regulator of Egr3 based on luciferase activity assays in RBL2H3 cells (**Figure S3A**). Antigen stimulation decreased miR-182-5p expression in RBL2H3 cells (**Figure S3B**). MiR-182-5p mimic inhibited increases of β-hexosaminidase activity (**Figure S3C**) and the expression levels of EGR3, HDAC6, TLR2, TLR4, and COX2 in RBL2H3 cells (**Figure S3D**) induced by antigen. MiR-182-5p mimic inhibited interactions of FcϵRI with Lyn and HDAC3 in RBL2H3 cells (**Figure S3D**) induced by antigen stimulation. However, Egr3 did not affect the expression of miR-182-5p (data not shown). Thus, miR-182-5p functions upstream of Egr3 and negatively regulates allergic inflammation *in vitro*.

### MiR-182-5p Negatively Regulates Anaphylaxis

Since miR-182-5p inhibited allergic inflammation *in vitro*, we next examined whether miR-182-5p could affect anaphylaxis. MiR-182-5p mimic exerted a negative effect on PCA in BALB/C mice (**Figure S4A**). MiR-182-5p mimic inhibited increase of β-hexosaminidase activity in BALB/C mice induced by PCA (**Figure S4B**). PCA decreased miR-182-5p expression (**Figure S4B**) but increased Egr3 expression at the transcriptional level in BALB/C mice (**Figure S4B**). MiR-182-5p mimic inhibited increases of EGR3, HDAC6, TLR2, TLR4, and COX2 expression levels (**Figure S4C**) and interactions of FcϵRI with HDAC3 and Lyn in BALB/C mice induced by PCA (**Figure S4C**). MiR-182-5p mimic inhibited decrease of rectal temperature (**Figure S5A**) and increase of β-hexosaminidase activity in BALB/C mice induced by PSA (**Figure S3B**). PSA decreased the expression of miR-182-5p in BALB/C mice (**Figure S5B**). MiR-182-5p mimic inhibited increases of EGR3, HDAC6, and HDAC3 expression levels in BALB/C mice induced by PSA (**Figure S5C**). Immunohistochemical staining employing lung tissues showed that miR-182-5p mimic inhibited increase of EGR3 expression in BALB/C mice induced by PSA (**Figure S5D**). These results indicate that miR-182-5p can inhibit anaphylaxis by regulating expression levels of EGR3 and HDAC6.

### IL-27 Increased by Antigen Stimulation in an HDAC6-Dependent Manner Mediates Allergic Inflammation *In Vitro*

**Figure 5:**
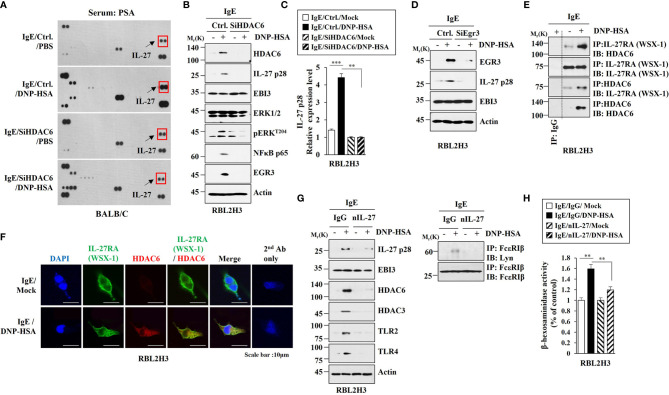
IL-27, increased in an HDAC6-dependent manner, mediates allergic inflammation *in vitro*. **(A)** BALB/C mice were intravenously injected with indicated siRNA (each at 3 μg/kg). The next day, BALB/C mice were intravenously injected with IgE. The following day, BALB/C mice were given an intravenous injection with DNP-HSA for 1 h. Serum from PSA-activated BALB/C mouse was subjected to cytokine array analysis (n=3). Representative image is shown. **(B)** RBL2H3 cells transfected with the indicated siRNA were sensitized with IgE for 24 h, and then stimulated by DNP-HSA for 1 h. Representative blots of three independent experiments are shown. **(C)** QRT-PCR was performed. **p<0.01; ***p<0.001. Average values of three independent experiments are shown. **(D)** Same as **(B)** except that cells were transfected with Egr3 siRNA. **(E)** Cell lysates from antigen-stimulated RBL2H3 cells were subjected to immunoprecipitation. Representative blots of three independent experiments are shown. **(F)** Immunofluorescence staining was performed. **(G)** The IgE-sensitized RBL2H3 cells were preincubated with the indicated antibody (each at 10 μg/ml) for 2 h, and then stimulated by DNP-HSA for 2 h. nIL-27 denotes neutralizing IL-27 antibody. Representative blots of three independent experiments are shown. **(H)** The β-hexosaminidase activity assays were performed. **p<0.01. Average values of three independent experiments are shown.

Since HDAC6 mediated anaphylaxis, it was necessary to identify downstream targets of HDAC6. Cytokine array analysis was performed to identify HDAC6-regualted cytokines. HDAC6 was necessary for the increase of IL-27 expression in BALB/C mouse model of PSA. (**Figure 5A**). The decrease of HDAC6 expression by RNA interference inhibited increases of EGR3, IL-27 p28, pERK^T204^, and NF-kB p65 expression levels in RBL2H3 cells induced by antigen stimulation (**Figure 5B**). Antigen stimulation did not affect expression level of EBI3, another subunit of IL-27 (**Figure 5B**). HDAC6 mediated the effect of antigen on the increase of IL-27 p28 expression at the transcriptional level in RBL2H3 cells (**Figure 5C**). The decrease of Egr3 expression by RNA interference inhibited increase of IL-27 p28 expression in RBL2H3 cells induced by antigen stimulation (**Figure 5D**). However, the decrease of Egr3 did not affect EBI3 expression regardless of antigen stimulation (**Figure 5D**). Antigen increased the interaction between IL-27 receptor alpha (WSX-1) and HDAC6 in RBL2H3 cells (**Figure 5E**). IL-27RA showed a co-localization with HDAC6 in antigen-stimulated RBL2H3 cells (**Figure 5F**). Blocking of IL-27 p28 by a neutralizing antibody (nIL-27 Ab) inhibited increases of IL-27 p28, HDAC3, HDAC6, TLR2, and TLR4 expression levels in RBL2H3 cells induced by antigen stimulation (**Figure 5G**). However, blocking of IL-27 p28 did not affect EBI3 expression level in RBL2H3 cells regardless of antigen stimulation (**Figure 5G**). IL-27 was necessary for antigen-induced interaction between FcϵRI and Lyn in RBL2H3 cells (**Figure 5G**). Blocking of IL-27 inhibited increase of β-hexosaminidase activity in RBL2H3 cells induced by antigen stimulation (**Figure 5H**). These results indicate a role of IL-27 in allergic inflammation. These results also suggest that IL-27 might induce features of allergic inflammation in an antigen-independent manner. The mouse recombinant IL-27 protein increased β-hexosaminidase activity (**Figure S6A**) and pERK^T204^, NFkBp65, EGR3 and HDAC6 expression levels in RBL2H3 cells in an antigen-independent manner (**Figure S6B**). Mouse recombinant IL-27 protein did not affect EBI3 expression level (**Figure S6B**). Mouse recombinant IL-27 protein induced interactions of HDAC6 with MyD88 and IL-27RA in RBL2H3 cells in an antigen-independent manner (**Figure S6B**). The decrease of IL-27 expression by RNA interference inhibited increases of IL-27 p28, HDAC6, EGR3, and pERK^T204^ expression levels in RBL2H3 cells induced by antigen stimulation (**Figure S6B**). The decrease of IL-27 expression did not affect EBI3 expression level (**Figure S6B**). The inhibition of NF-kB p65 by BAY-11-7082 prevented antigen from increasing expression levels of EGR3, HDAC6, and IL-27 p28 in RBL2H3 cells (**Figure S6B**). However, inhibition of NF-kB p65 did not affect EBI3 expression level in RBL2H3 cells (**Figure S6B**). Thus, IL-27 p28 and NF-kB p65 may form a positive feedback loop. The decrease of EBI3 prevented antigen from increasing expression levels of IL-27p28, HDAC6, and EGR3 in RBL2H3 cells (**Figure S6C**). Mouse recombinant IL-27 protein increased vascular permeability and β-hexosaminidase activity in BALB/C mice in an antigen-independent manner (**Figure S6D**). Mouse recombinant IL-27 protein also increased IL-27 p28 mRNA expression level in an antigen-independent manner (**Figure S6D**). Mouse recombinant IL-27 protein increased expression levels of hallmarks of allergic inflammation (**Figure S6E**) and induced interactions of FcϵRI with HDAC3 and Lyn in BALB/C mice in an antigen-independent manner (**Figure S6E**). However, mouse recombinant IL-27 protein did not affect EBI3 expression level (**Figure S6E**). These results imply that IL-27 can indeed mediate allergic inflammation both *in vitro* and *in vivo*.

### IL-27 Mediates Anaphylaxis

**Figure 6:**
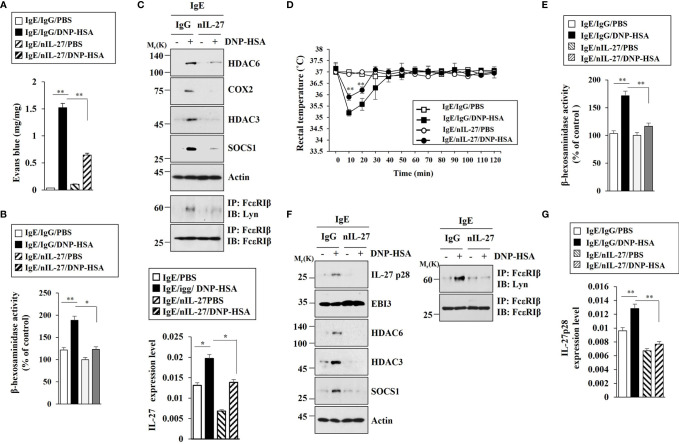
IL-27 mediates both PCA and PSA. **(A)** BALB/C mice were given an intradermal injection of IgE (0.5 μg/kg) and an intravenous injection of the indicated antibody (each at 20 µg/kg). The next day, BALB/C mice were intravenously injected with PBS or DNP-HSA (250 μg/kg) along with 2% (v/v) Evans blue solution. Each experimental group comprised four BALB/C mice. **p<0.01. **(B)** The β-hexosaminidase activity assays using ear tissue lysates were performed (n=4). *p<0.05; **p<0.01. Average values of three independent experiments are shown. **(C)** Immunoblot and immunoprecipitation employing ear tissue lysates were performed (n=4). Representative blots of three independent experiments are shown. Ear tissue lysates were also subjected to qRT-PCR (lower). *p<0.05. **(D)** BALB/C mice were given an intravenous injection of DNP-specific IgE (0.5 µg/kg) along with the indicated antibody (each at 20 µg/kg). The next day, BALB/C mice were given an intravenous injection with DNP-HSA (250 μg/kg), and the rectal temperatures were measured. Each experimental group comprised four mice. **p<0.01, compared with IgE/IgG/DNP-HSA. **(E)** The β-hexosaminidase activity assays employing lung tissue lysates were performed (n=4). **p<0.01. Average values of three independent experiments are shown. **(F)** Immunoblot and immunoprecipitation employing lung tissue lysates were performed (n=4). Representative blots of three independent experiments are shown. **(G)** QRT-PCR analysis employing lung tissue lysates was performed. **p<0.01. Average values of three independent experiments are shown.

Since IL-27 promoted features of allergic inflammation in an antigen-independent manner, we examined the role of IL-27 in anaphylaxis. Blocking of IL-27 p28 by neutralizing antibody (nIL-27) exerted a negative effect on vascular permeability in BALB/C mouse model of PCA (**Figure 6A**). Blocking of IL-27 p28 inhibited increase of β-hexosaminidase activity (**Figure 6B**) and increases of HDAC6, HDAC3, SOCS1, and COX2 expression levels in BALB/C mice induced by PCA (**Figure 6C**). Blocking of IL-27 inhibited interaction between FcϵRI and Lyn in BALB/C mice induced by PCA (**Figure 6C**). PCA increases IL-27 expression at the transcriptional level (**Figure 6C**). IL-27 mediated PSA (**Figure 6D**). Blocking of IL-27 inhibited increase of β-hexosaminidase activity (**Figure 6E**) and IL-27 p28, HDAC6, HDAC3, and SOCS1 expression levels in BALB/C mice induced by PSA (**Figure 6F**). Blocking of IL-27 did not affect EBI3 expression in BALB/C mice (**Figure 6F**). Blocking of IL-27 inhibited interaction between FcϵRI and Lyn in BALB/C mice induced by PSA (**Figure 6F**). PSA increased IL-27 p28 expression at the transcriptional level in BALB/C mice (**Figure 6G**). It has been shown that allergic inflammation can enhance tumorigenic and metastatic potentials of cancer cells (7). Thus, IL-27 might enhance tumorigenic and metastatic potentials of cancer cells.

### HDAC6 Is Necessary for Tumorigenic and Metastatic Potential Enhanced by PSA

**Figure 7:**
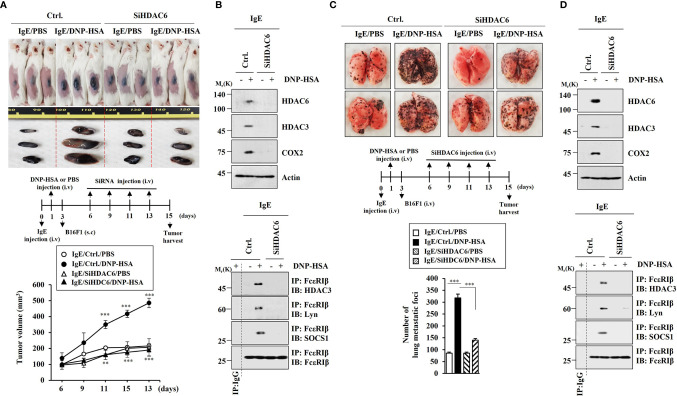
HDAC6 is necessary for tumorigenic and metastatic potential enhanced by PSA. **(A)** PSA was induced as described. Each mouse was given an injection with B16F1 melanoma cells (2 × 10^5^) on day 3 of the time line. After tumor reached a certain size, each BALB/C mouse was intravenously injected with the indicated siRNA (each at 3 μg/kg) on the indicated days. Each experimental group comprised four BALB/C mice. **p<0.01, compared with IgE/Ctrl./DNP-HSA; ***p<0.001, compared with IgE/Ctrl./PBS **(B)** Immunoblot and immunoprecipitation employing tumor tissue lysates were performed (n=4). Representative blots of three independent experiments are shown. **(C)** Each mouse was injected with B16F1 melanoma cells (2 × 10^5^) on day 3. The number of lung metastatic foci was determined. Each experimental group comprised four BALB/C mice. ***p<0.001. **(D)** Immunoblot and immunoprecipitation employing tumor lysates were performed (n=4). Representative blots of three independent experiments are shown.

Since PSA could enhance tumorigenic and metastatic potential (7), we examined whether HDAC6 was needed for the tumorigenic and metastatic potentials enhanced by PSA. RNA interference of HDAC6 showed that HDAC6 was necessary for tumorigenic (**Figure 7A**) and metastatic potential (**Figure 7C**) enhanced by PSA. RNA interference of HDAC6 also showed that HDAC6 mediated increases of allergic inflammation hallmarks and interactions of FcϵRI with HDAC3, Lyn, and SOCS1 induced by PSA (**Figures 7B, D**). These results imply that IL-27 might be necessary for cellular interactions important for tumorigenic and metastatic potentials enhanced by PSA.

### IL-27 Is Necessary for Enhanced Metastatic Potential by PSA

**Figure 8:**
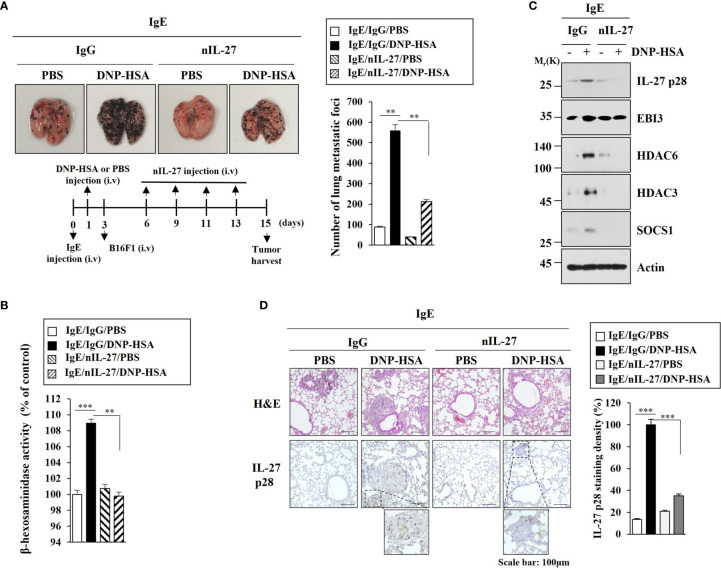
IL-27 is necessary for enhanced metastatic potential by PSA. **(A)** Each mouse was injected with B16F1 melanoma cells (2 × 10^5^) on day 3. The extent of lung metastasis was determined. **p<0.01. **(B)** The β-hexosaminidase activity assays employing lung tumor tissue lysates were performed (n=4). **p<0.01; ***p<0.001. Average values of three independent experiments are shown. **(C)** Immunoblot employing tumor lysates was performed (n=4). Representative blots of three independent experiments are shown. **(D)** Lung tumor tissues were subjected to H&E staining and Immunohistochemical staining. Representative images of the staining are shown (n=3). Quantification was performed using Image J (NIH). ***p<0.001.

Since HDAC6 was necessary for the metastatic potential enhanced by PSA, we examined the effect of IL-27 on the metastatic potential of cancer cells. PSA enhanced metastatic potential of mouse melanoma B16F1 cells (**Figure 8A**) and increased β-hexosaminidase activity (**Figure 8B**) in an IL-27-dependent manner. Immunoblot of lung tumor tissue lysates showed that PSA increased expression levels of HDAC6, HDAC3, and SOCS1 in an IL-27-dependent manner (**Figure 8C**). However, PSA did not affect EBI3 expression (**Figure 8C**). Immunohistochemical staining employing lung tumor tissue showed that the enhanced metastatic potential of B16F1 cells was accompanied by the increased expression of IL-27 p28 (**Figure 8D**). These results led us to hypothesize that IL-27 might mediate cellular interactions that are necessary for tumorigenic and metastatic potentials enhanced by PSA.

### IL-27 Is Present in Exosomes and Mediates Cellular Interactions During Allergic Inflammation

**Figure 9:**
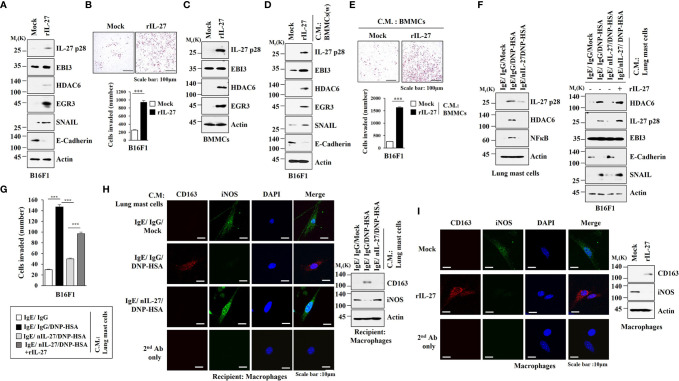
IL-27 is necessary for enhanced invasion of B16F1 melanoma cells and lung macrophages polarization induced by mast cells. **(A)** B16F1 cells were treated without or with mouse recombinant IL-27 protein (20 ng) for 1 h. rIL-27 denotes mouse recombinant IL-27 protein. Representative blots of three independent experiments are shown. **(B)** Invasion of B16F1 cells was determined. ***p<0.001. Average values of three independent experiments are shown. **(C)** BMMCs were treated without or with IL-27 protein (20 ng/ml) for 1 h. Representative blots of three independent experiments are shown. **(D)** Culture medium of BMMCs treated with mouse recombinant IL-27 protein (20 ng/ml) for 2 h was removed and replaced with serum-free DMEM. At 12 h after, culture medium was added to B16F1 cells for 16 h. W denotes removal of culture medium of the cells treated with mouse recombinant IL-27 protein. Representative blots of three independent experiments are shown. **(E)** Invasion of B16F1 cells was determined. ***p<0.001. Average values of three independent experiments are shown. **(F)** Lung mast cells were treated with DNP-specific IgE along with the indicated antibody (each at 10 μg/ml) for 24 h, and stimulated by DNP-HSA along without or with rIL-27 (20 ng/ml) for 1 h (left). The culture medium was then added to B16F1cells for 16 h (right). **(G)** Same as **(F)** except that invasion of B16F1 cells was determined. ***p<0.001. Average values of three independent experiments are shown. **(H)** Lung mast cells were treated with DNP-specific IgE along with the indicated antibody (each at 10 μg/ml) for 24 h, and then stimulated by DNP-HSA for 1 h. The culture medium was then added to lung macrophages for 16 h. Immunofluorescence staining (left) and immunoblot (right) were performed. Representative blots of three independent experiments are shown. **(I)** Lung macrophages were treated without or with mouse recombinant IL-27 protein (20 ng/ml) for 1 h. Immunofluorescence staining (left) and immunoblot (right) were performed. Representative blots of three independent experiments are shown.

**Figure 10:**
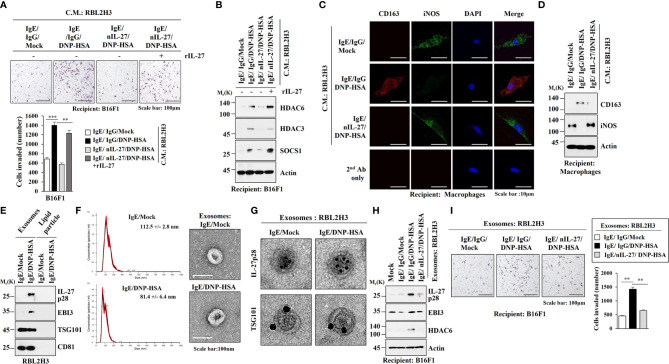
IL-27 is present in exosomes and mediates the effects of exosomes of RBL2H3 cells on invasion of B16F1 melanoma cells. **(A)** RBL2H3 cells were treated with DNP-specific IgE along with the indicated antibody (each at 10 μg/ml) for 24 h, and then stimulated by DNP-HSA along with rIL-27 (20 ng/ml) for 1 h. The culture medium was then added to B16F1cells for 16 h. Invasion potential of B16F1 cells was determined. **p<0.01; ***p<0.001. Average values of three independent experiments are shown. **(B)** Immunoblot employing B16F1 cell lysates was performed. Representative blots of three independent experiments are shown. **(C)** RBL2H3 cells were treated with DNP-specific IgE along with the indicated antibody (each at 10 μg/ml) for 24 h, and then stimulated by DNP-HSA. Culture medium was then added to lung macrophages for 16 h, followed by immunofluorescence staining. **(D)** Immunoblot was performed. Representative blots of three independent experiments are shown. **(E)** Exosomes isolated from RBL2H3 cells were subjected to immunoblot. Representative blots of three independent experiments are shown. **(F)** Shows size distributions of exosomes isolated from RBL2H3 cells employing nanoparticle tracking analysis (NTA). Exosomes isolated from RBL2H3 cells were observed by negative staining electron microscopy (right). **(G)** Immuno-gold staining images using anti-TSG101, a known membrane marker for the exosomes, and anti-IL-27 p28 antibody. Twenty-five and 10 nm gold particles show the presence of TSG101 and IL-27 p28, respectively. **(H)** Exosomes (20 μg) isolated from RBL2H3 cells were added to B16F1 cells for 24 h. immunoblot was performed. Representative blots of three independent experiments are shown. **(I)** Invasion assays were performed as described. **p<0.01. Average values of three independent experiments are shown.

It is known that enhanced tumorigenic and metastatic potential of cancer cells results from cellular interactions between cancer cells and immune cells such as mast cells and macrophages (7). We first examined whether IL-27 could affect invasion potential of cancer cells. Mouse recombinant IL-27 protein increased expression levels of IL-27p28, HDAC6, EGR3 and SNAIL in B16F1 cells (**Figure 9A**) and enhanced invasion of B16F1 cells (**Figure 9B**). Mouse recombinant IL-27 protein did not affect EBI3 expression level in B16F1 cells (**Figure 9B**). Mouse recombinant IL-27 protein also increased expression levels of HDAC6 and EGR3 in BMMCs in an antigen-independent manner (**Figure 9C**). The culture medium of BMMCs treated with mouse recombinant IL-27 protein increased expression levels of EGR3 and SNAIL in B16F1 cells (**Figure 9D**) and enhanced the invasion of B16F1 cells (**Figure 9E**). Culture medium of BMMCs treated with mouse recombinant IL-27 protein for 2 h was washed and replaced with serum-free DMEM. At 12 h after, culture medium was then added to B16F1 cells (**Figure 9D**). Thus, the effect of culture medium of BMMCs on the increased expression levels of EGR3 and SNAIL in B16F1 cells may not be a direct effect of recombinant IL-27 protein in the culture medium.The above results suggest that mouse recombinant IL-27 protein can promote cellular interaction in an antigen-independent manner. Antigen increased HDAC6 expression in an IL-27-dependent manner in lung mast cells (**Figure 9F**, left). Culture medium of lung mast cells increased expression levels of HDAC6 and SNAIL, but decreased the expression level of E-cadherin in an IL-27-dependent manner in B16F1 cells (**Figure 9F**, right). Culture medium of lung mast cells enhanced the invasion of B16F cells in an IL-27-dependent manner (**Figure 9G**). Culture medium of lung mast cells increased CD163 expression, but decreased inducible nitric oxide synthase (iNOS) expression in lung macrophages in an IL-27-dependent manner (**Figure 9H**). Mouse recombinant IL-27 protein increased CD163 expression, but decreased iNOS expression in lung macrophages (**Figure 9I**). CD163 and iNOS are markers M2 macrophages and M1 macrophages, respectively. Since culture medium of BMMCs treated with mouse recombinant IL-27 protein enhanced the expression levels of EGR3 and SNAIL (**Figure 9D**) and the invasion of B16F1 cells (**Figure 9E**), we first examined whether macrophages polarization induced by culture medium of mast cells was a direct effect of the recombinant IL-27 protein in the culture medium. For this, culture medium of BMMCs treated with mouse recombinant IL-27 protein for 2 h was removed and replaced with serum-free DMEM. At 12 h after, culture medium was added to lung macrophages. Thus obtained culture medium increased CD163, but decreased iNOS expression in lung macrophages (**Figure S7A**). This suggests that effect of culture medium of BMMCs on macrophages polarization was not a direct effect of the recombinant IL-27 protein in the culture medium. The effects of culture medium of B16F1 cells on mast cell activation and M2 macrophages were not a direct effect of the recombinant IL-27 protein in the culture medium (**Figure S7B**). Effects of culture medium of lung macrophages on the increased expression levels of IL-27p28, HDAC6, and EGR3 in B16F1 cells and BMMCs were not a direct effect of the recombinant IL-27 protein in the culture medium (**Figure S7C**). These results imply that IL-27 can mediate cellular interactions necessary for tumorigenic and metastatic potentials enhanced by allergic inflammation. Further studies are needed to identify downstream targets of IL-27 for better understanding of cellular interactions during allergic inflammation. We next examined whether exosomes could mediate these cellular interactions. Culture medium of RBL2H3 cells enhanced the invasion of B16F1 cells in an IL-27-dependent manner (**Figure 10A**) and increased allergic inflammation hallmarks including HDAC6, HDAC3, and SOCS1 in an IL-27-dependent manner in B16F1 cells (**Figure 10B**). Culture medium of RBL2H3 cells increased CD163 expression but decreased iNOS expression in an IL-27-dependent manner in lung macrophages (**Figures 10C, D**). These results imply that exosomes might mediate these cellular interactions during allergic inflammation. We also hypothesized that exosomes contained IL-27. Immunoblot of exosomes from antigen-stimulated RBL2H3 cells showed the presence of IL-27 p28 and EBI3 (**Figure 10E**). Exosomes isolated from RBL2H3 cells displayed normal sizes based on nanoparticle tracking analysis (NTA) (**Figure 10F**). Exosomes were seen in the culture medium of RBL2H3 cells regardless of antigen stimulation (**Figure 10F**, right). Immuno-EM showed the presence of IL-27 p28 within exosomes of antigen-stimulated RBL2H3 cells (**Figure 10G**). Exosomes of antigen-stimulated RBL2H3 cells increased HDAC6 expression (**Figure 10H**) and enhanced the invasion of B16F1 cells (**Figure 10I**) in an IL-27-dependent manner. PKH67-labeling of exosomes showed internalization of exosomes from RBL2H3 cells into B16F1 cells and lung macrophages (**Figures S8A, B**). Exosomes of RBL2H3 cells increased CD163 expression, but decreased iNOS expression in an IL-27-dependent manner in lung macrophages (**Figure S8C**). These results indicate that IL-27-mediated cellular interactions might be responsible for remodeled tumor microenvironment induced by allergic inflammation. Exosomes of PSA-activated mast cells might enhance the invasion of cancer cells and induce M2 macrophages polarization.

## Discussion

Egr3 was found to be one of the most highly upregulated genes in antigen-stimulated RBL2H3 cells. Egr3 is an immediate early zinc finger transcriptional factor activated by mitogenic signals (33). Egr3 promotes an adaptive immune response by driving Th17 response (34). We have previously reported the role of SOCS1 in allergic inflammation both *in vitro and in vivo* (2). Egr3 can directly induce the expression of SOCS1 (35). Egr3 might regulate Th17/Treg balance during allergic inflammation. Further studies are needed to identify Egr3-regualted cytokines that mediate anaphylaxis.

NF-κB and MAPK signaling pathways can regulate Egr3 expression in breast adipose fibroblasts (36). NF-κB signaling is critical for mast cell-mediated allergic inflammation (37). Eupatilin can suppress NF-κB-mediated anaphylactic shock and the release of histamine by promoting phosphorylation and degradation of IκBα *via* the Akt/IKK(α/β) pathway (38). Our results showed that antigen stimulation increased NF-κB p65 expression in RBL2H3 cells. ChIP assays showed that NF-κB p65 could bind to the promoter sequences of Egr3. We showed that inhibition of NF-κB p65 exerted a negative effect on increases of Egr3 expression in RBL2H3 cells induced by antigen stimulation. Inhibition of Egr3 decreases NF-κB activity during myoblast proliferation (39). Decreased expression of Egr3 by RNA interference prevented antigen from increasing NF-κB p65 expression in RBL2H3 cells. Thus, Egr3 and NF-κB p65 form a positive feedback loop to regulate allergic inflammation. The mechanism involved in the increase of NF-κB p65 expression during allergic inflammation merits further study. NF-κB might play a critical role in anaphylaxis.

House dust mite allergen Derp5 can increase IL-8 expression in respiratory epithelial cells through NF-κB signaling (40). IL-6 and IL-8 function as downstream targets of Egr3 in breast cancers (8). Therefore, Egr3 may increase expression levels of IL-6 and IL-8 during anaphylaxis *via* NF-κB. Further studies are needed to examine effects of IL-6 and IL-8 on allergic inflammations *in vitro* and *in vivo*.

HDAC6 can mediate allergic airway inflammation by increasing expression levels of IL-4 and IL-5 (17). It is known that HDAC6 can promote CD8 T cell activation during allergic skin inflammation (41), mediate the activation of TGFβ-Notch signaling (42), and mediate airway hyperresponsiveness in obese mice with asthma (43). Notch1 can mediate asthma by regulating Th1/Th2 balance (44). These reports imply the role of HDAC6 in allergic inflammations such as anaphylaxis. Our results showed roles of HDAC6 in PSA and allergic inflammation *in vitro*. Global identification of downstream targets of HDAC6 is needed in the future to better understanding the mechanism of HDAC6-promoted anaphylaxis.

MiR-182-5p can inhibit oxidative stress and apoptosis by inactivating TLR4 in atherosclerosis (45). It is known that miR-182-5p targets TLR4 and attenuates ischemia-reperfusion injury (46). MiR-182-5p can decrease TLR4 expression by binding to its 3’-UTR (47). We found that TLR4 was necessary for both PCA and PSA (personal observations). We therefore hypothesized that miR-182-5p might regulate the expression of Egr3. TargetScan analysis predicted that miR-182-5p was a negative regulator of Egr3. We found that miR-182-5p could bind to the 3’-UTR of Egr3. Our results showed regulatory effects of miR-182-5p on PCA and PSA. Further studies are needed to identify more targets of miR-182-5p.

HDAC6 can increase expression levels of proinflammatory genes *via* ROS-MAPK-NF-κB/AP-1 pathways (48). Our results showed that Egr3 and HDAC6 were necessary for increased expression levels of pERK^T204^ and NF-kB p65 in antigen-stimulated RBL2H3 cells. Cytokine array analysis showed that HDAC6 was responsible for the increase of IL-27 expression in a mouse model of PSA. HDAC6 was also responsible for the increase of IL-27 RNA level in antigen-stimulated RBL2H3 cells.

TLR7/8 agonist R848 suppresses experimental asthma by increasing IL-27 level (49). IL-27 receptor (WSX-1) suppresses mast cell activity (50). IL-27 directly inhibits Th2 cytokine production (50). These reports suggest anti-inflammatory effect of IL-27. However, cytokines can display dual roles. In this study, we found that antigen stimulation increased IL-27 p28, but not EBI3, another subunit of IL-27. QRT-PCR analysis also showed that antigen stimulation did not affect EBI3 mRNA level in RBL2H3 cells or BALB/C mouse model of PCA or PSA (personal observations). The role of EBL3 in anaphylaxis remains to be seen.

IL-27 can increase levels of IL-6 and IL-8 by activating TLR4-JAK signaling pathways (51). Further studies are needed to examine the effect of HDAC6 on the JAK signaling pathways. IL-27, along with IFN-γ, can mediate virus-induced allergic airway inflammation (52). TLR3/4 and IFNAR signaling pathways are known to regulate the production of IL-27 (53). TLR4 activation by lipopolysaccharide (LPS) can increase the expression of IL-27 in macrophages (53). IL-27 induces the activation of ERK and plays a pro-inflammatory role (54). IL-27 is involved in steroid-resistant airway hyperresponsiveness (55). IL-27 amplifies airway inflammation by increasing CXCL10 production (56). These reports imply roles of IL-27 in anaphylaxis. HDAC6 might regulate transcriptional factors that directly regulate IL-27 expression. Further studies are needed to identify transcriptional factors for better understanding the mechanism involved in IL-27-medaited anaphylaxis.

We found that mouse recombinant IL-27 protein promoted features of allergic inflammation both *in vitro* and *in vivo* in an antigen-independent manner. This implies that IL-27 can mediate cellular interactions during allergic inflammation. Our results showed roles of IL-27 in anaphylaxis and metastatic potential of cancer cells enhanced by PSA. Cellular interactions are responsible for tumorigenic and metastatic potentials enhanced by PSA (7). Thus, IL-27 may mediate these cellular interactions. High level of interleukin-27 is seen in patients with lymph node metastatic gastroesophageal cancer (57). IL-27 promotes survival, decreases apoptosis and sensitivity to chemotherapy in AML cells (58). IL-27 induces the expression of immune-regulatory molecules such as PD-L1 and IDO in human ovarian cancer cells (59). IL-27 increases PDL1 expression in human lymphoma macrophages (60). CD63 suppresses hepatocellular carcinoma by inhibiting the increase of IL-27 (61). IL-27 increases CD39 expression in ovarian cancer associated macrophages (62). B16F10 melanoma cells that express single chain IL-27 exerts anti-angiogenic and antitumor activity (63). These anti-angiogenic and antitumor activities were observed in IFN-γ knockout mice (63). In this study, we showed that IL-27 was necessary for metastatic potential of B16F1 cells enhanced by PSA. IL-27 is necessary for nitric oxide (NO) production induced by LPS in peritoneal macrophages (64). IL-27 negatively regulates iNOS-producing dendritic cells (65). We showed that IL-27 was responsible for the decrease of iNOS in lung macrophages by antigen-stimulated lung mast cells.

Further studies are needed to examine the role of IL-27 in remodeled tumor microenvironment induced by cellular interactions involving cancer cell and immune cells such as mast cells and macrophages. Experiments employing culture medium showed that IL-27 was involved in cellular interactions responsible for tumorigenic and metastatic potentials enhanced by allergic inflammation. Further studies are needed identify IL-27-regualted molecules for better understanding the mechanism involved in tumorigenic and metastatic potentials enhanced by allergic inflammation.

Exosomes can mediate cellular interactions during allergic inflammation (7). It is known that enhanced secretion of exosomes by epithelial cells contributes to the pathogenesis of asthma in an IL-13-dependent manner (66). Mast cells can secrete exosomes upon FcϵRI engagement (67). Mast cell-derived exosomes can induce epithelial mesenchymal transition in epithelial cells (68). Exosomes are known to mediate cellular interactions of cancer cells, macrophages, and mast cells in the tumor microenvironment (69). We found that exosomes of antigen-stimulated RBL2H3 cells enhanced the invasion of B16F1 melanoma cells and induced M2 macrophages polarization in an IL-27-dependent manner. We also found the presence of IL-27 in exosomes of antigen-stimulated RBL2H3 cells. Exosomes of PSA-activated mast cells and macrophages might contain IL-27. Thus, exosomes of PSA-activated mast cells and macrophages may enhance tumorigenic and metastatic potential of cancer cells. Further studies are needed to identify targets of these exosomes. Identification of more exosomal molecules can lead to better understanding of the cellular interactions during allergic inflammation.

RNA sequencing analysis showed that expression levels of CCL1, CCL3, and CCL7 were increased by antigen stimulation in RBL2H3 cells (data not shown). FcϵRI activation in mast cells can increase expression levels of chemokines such as CCL1, CCL3, and CCL7 (70)). Increased CCL1 expression is necessary for the activation of mast cells (71). Egr2 is necessary for the increased expression of CCL1 induced by allergens (71). Human mast cells can release CCL2 *via* MAPK and NF-kB in response to IL-33 (72). Thus, CCL1 and CCL2 might mediate allergic inflammation. CCL7 mediates OVA-induced ocular anaphylaxis and mast cell activation *in vitro* (73). Further studies are needed to check whether EGR3-HDAC6-IL-27 axis could regulate expression levels of these chemokines.

In summary, this study showed novel roles of EGR3-HDAC6-IL-27 axis in cellular interactions that are necessary for allergic inflammation and tumorigenic and metastatic potentials enhanced by allergic inflammation (**Figure S9**). The EGR3-HDAC6-IL-27 axis can be employed as a target for developing anti-allergy therapeutics.

## Data Availability Statement

The datasets presented in this study can be found in online repositories. The names of the repository/repositories and accession number(s) can be found in the article/**Supplementary Material**.

## Ethics Statement

The animal study was reviewed and approved by Institutional Animal Care and Use Committee of Kangwon National University.

## Author Contributions

YKim, HJ, and DJ designed the study. YKwon, MK, and MJ performed the experiments. DJ wrote the manuscript. All authors contributed to the article and approved the submitted version.

## Funding

This work was supported by National Research Foundation Grants (2017M3A9G7072417, 2020R1A2C1006996, and 2018R1D1A1B07043498), a grant from the BK21 plus Program.

## Conflict of Interest

The authors declare that the research was conducted in the absence of any commercial or financial relationships that could be construed as a potential conflict of interest.

## Abbreviations

BMMC, bone marrow-derived mast cells; CCL1, CC chemokine ligand 1; COX2, cyclooxygenase 2; DNP-HSA, 2,4-dinitrophenyl human serum albumin; Egr3, Early growth response 3; ERK, extracellular regulated kinase; HDAC6, histone deacetylase 6; IL-27, Interleukin-27; iNOS, inducible nitric oxide synthase; miRNA, microRNA; Myd88, Myeloid differentiation primary response 88; PCA, passive cutaneous anaphylaxis; PSA, passive systemic anaphylaxis; RBL2H3, rat basophilic leukemia; SiRNA, small interfering RNA; SOCS1, suppressor of cytokine signaling 1; TLR, toll-like receptor; UTR, untranslated region.
