# Supplementary material for: EGR3-HDAC6-IL-27 Axis Mediates Allergic Inflammation and Is Necessary for Tumorigenic Potential of Cancer Cells Enhanced by Allergic Inflammation-Promoted Cellular Interactions
Source: Front Immunol. 2021 Jun 21;12:680441. doi: 10.3389/fimmu.2021.680441 (PMC8257050; doi:10.3389/fimmu.2021.680441)
Supplement: Supplementary file 1 [file DataSheet_1.docx]

Supplementary Figures


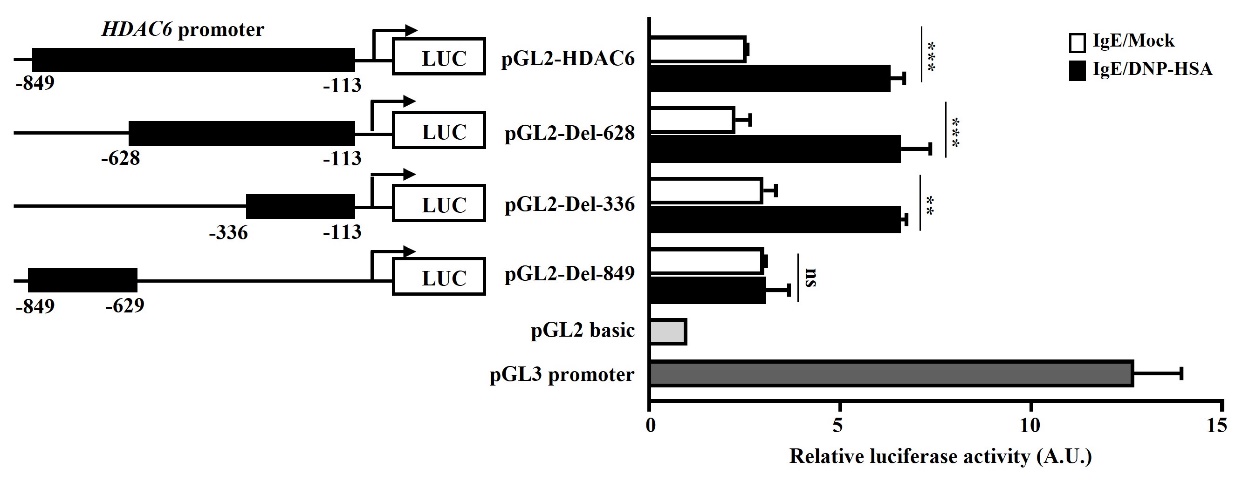


**Figure S1.** Antigen stimulation increases *HDAC6* promoter activity. The indicated promoter-luciferase construct was transiently transfected into RBL2H3 cells. At 24 hours after transfection, cells were sensitized with IgE for 24 h, followed by stimulation with DNP-HSA for 1 h. Luciferase activity assays were performed. Average values of three independent experiments are shown. P1 (-849~ -629), P2 (-628~ -337), and P3 (-336~ -113) denote primer-binding sites.


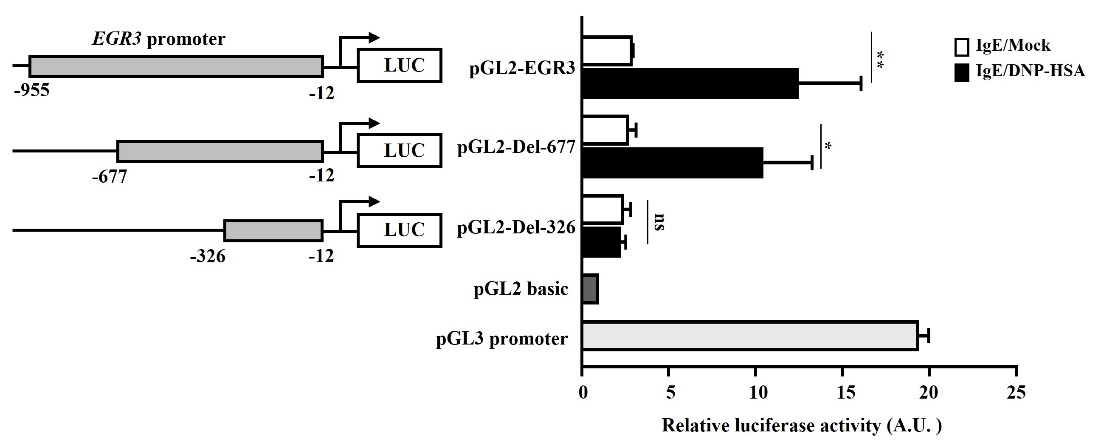


**Figure S2.** Antigen stimulation increases *Egr3* promoter activity. The indicated promoter-luciferase construct was transiently transfected into RBL2H3 cells. At 24 hours after transfection, cells were sensitized with IgE for 24 h, followed by stimulation with DNP-HSA for 1 h. Luciferase activity assays were performed. Average values of three independent experiments are shown. P1 (-955~ -678), P2 (-677~ -327), and P3 (-326~ -12) denote primer-binding sites.


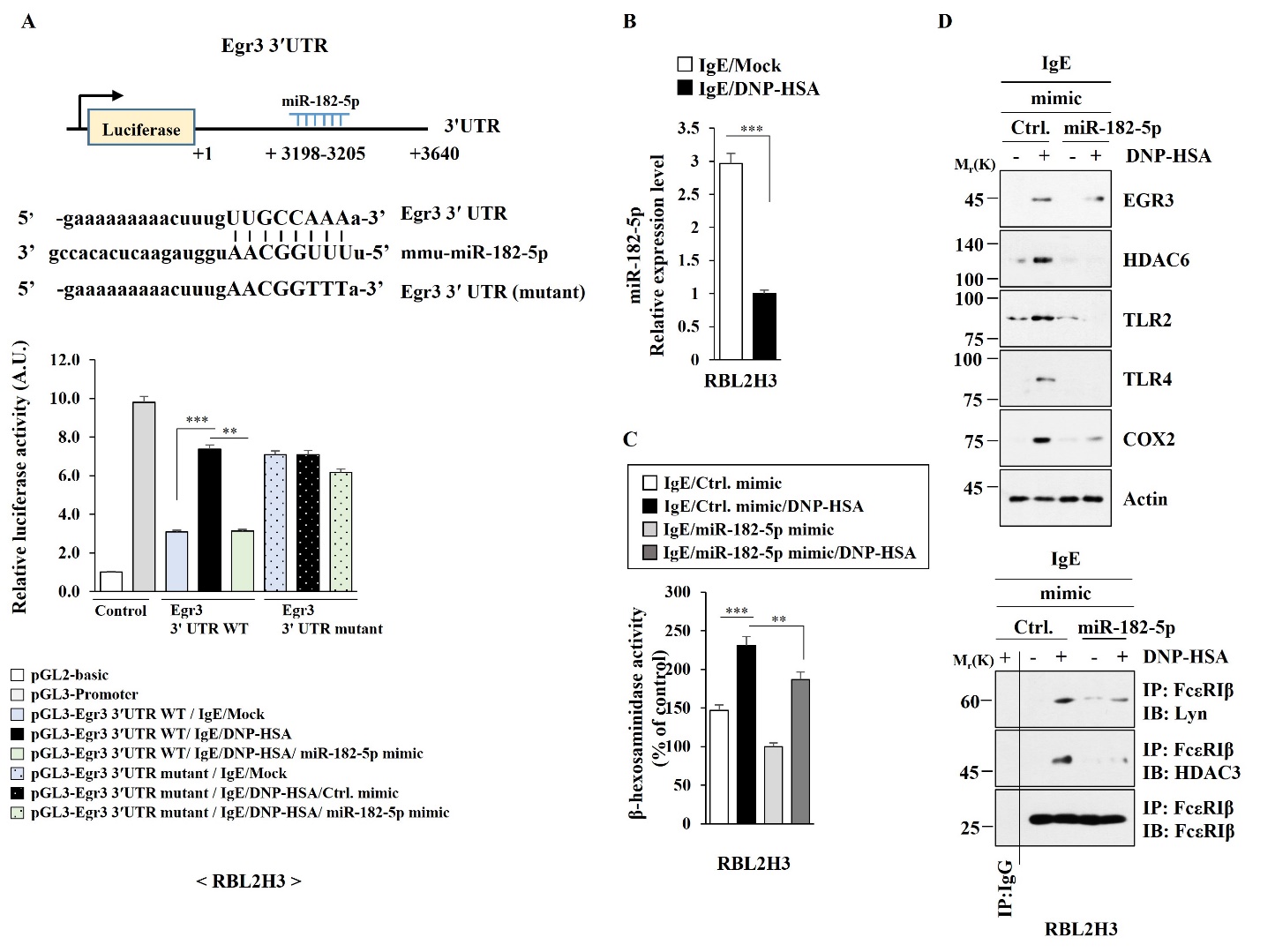


**Figure S3.** MiR-182-5p targets Egr3 and inhibits allergic inflammation *in vitro*. **(A)** The indicated cells were transfected with wild type Luc-Egr3′-UTR or mutant Luc-Egr3′-UTR along with the indicated mimic (each at 10 nM). At 48 h after transfection, luciferase activity assays were performed. **, *p*<0.01; ***, *p*<0.001. Average values of three independent experiments are shown. **(B)** QRT-PCR was performed in RBL2H3 cells treated without or with DNP-HSA (100 ng/ml) for 1 h. ***, *p*<0.001. Average values of three independent experiments are shown. **(C)** RBL2H3 cells transfected with the indicated mimic (each at 10 nM). The next day, cells were then sensitized with IgE for 24 h, and then stimulated without or with DNP-HSA for 1 h. The β-hexosaminidase activity assays were performed. **, *p*<0.01; ***, *p*<0.001. **(D)** Immunoblot and immunoprecipitation were performed. Representative blots of three independent experiments are shown.


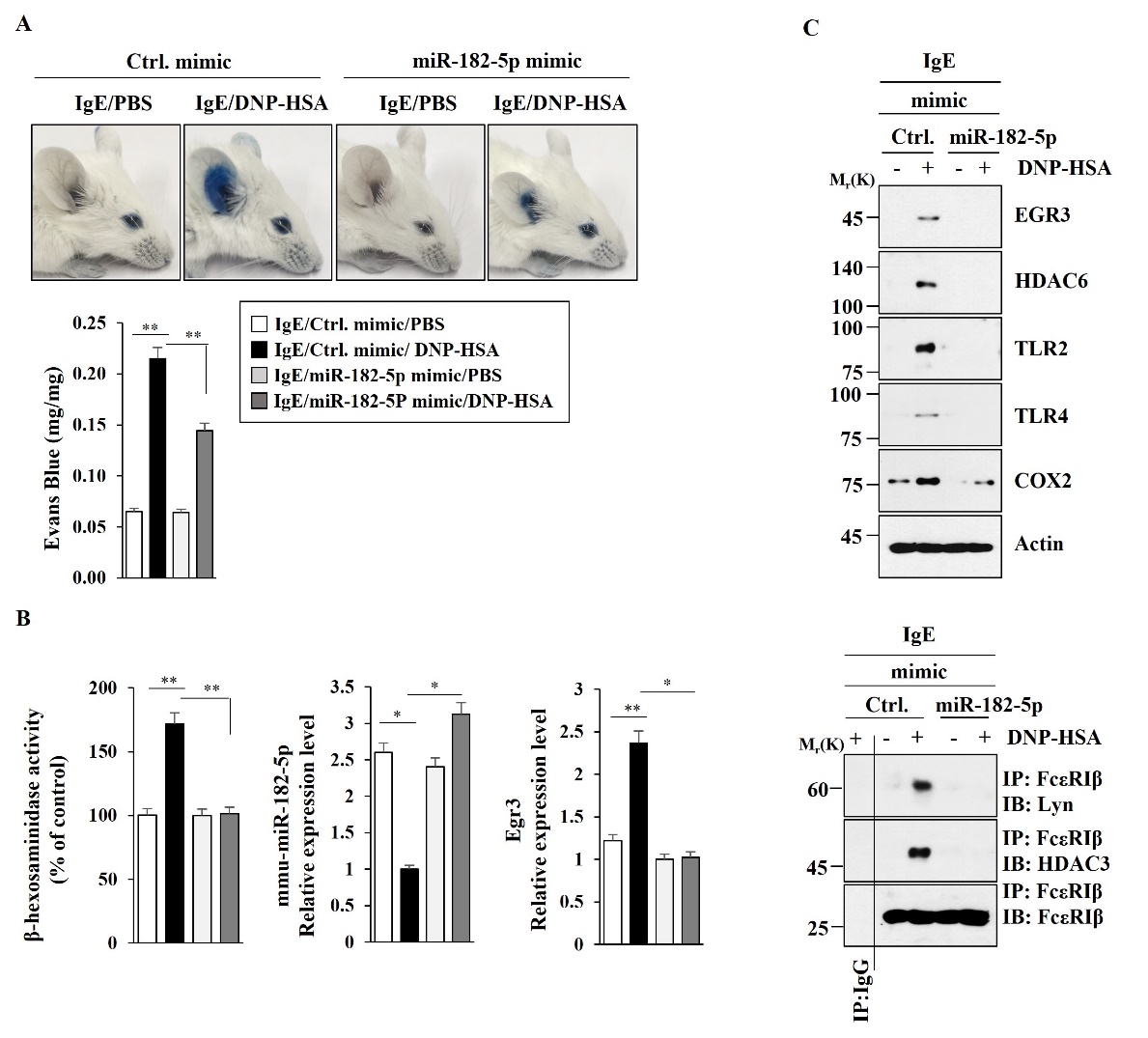


**Figure S4.** MiR-182-5p mimic inhibits PCA. **(A)** BALB/C mice were subjected to an intradermal injection of IgE (0.5 μg/kg) and an intravenous injection of the indicated mimic (each at 3 μg/kg). The next day, BALB/C mice were the intravenously injected with PBS or DNP-HSA (250 μg/kg) along with 2% (v/v) Evans blue solution. Each experimental group comprised four BALB/C mice. **, *p*<0.01. **(B)** Ear tissue lysates from BALB/C mouse of each experimental group were subjected to β-hexosaminidase activity assay (n=4) and qRT-PCR (n=4). *, *p*<0.05; **, *p*<0.01. Average values of three independent experiments are shown. **(C)** Immunoblot and immunoprecipitation of ear tissue lysates (n=4) were performed. Representative blots of three independent experiments are shown.


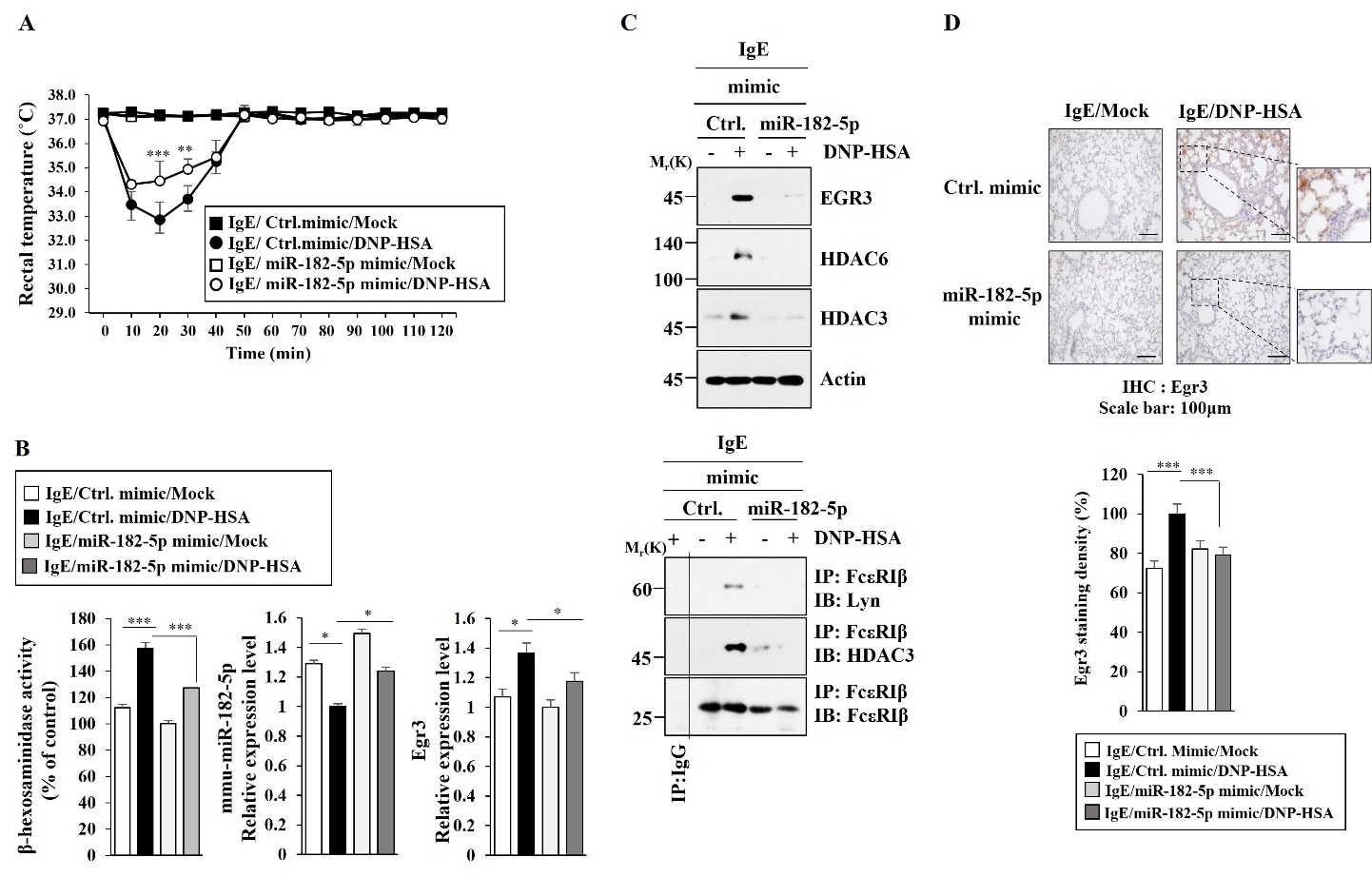


**Figure S5.** MiR-182-5p mimic inhibits PSA. **(A)** BALB/C mice were intravenously injected with the indicated mimic (each at 3 μg/kg). The next day, BALB/C mice were intravenously injected with IgE (0.5 μg/kg). The following day, BALB/C mice were then intravenously injected with DNP-HSA (250 μg/kg), and rectal temperatures were measured. Each experimental group comprised five mice. The means ± S.E. of three independent experiments are depicted. **, *p*<0.01; ***, *p*<0.001, compared with IgE/Ctrl.mimic/DNP-HSA. **(B)** The β-hexosaminidase activity assays and qRT-PCR analysis employing lung tissues were performed (n=4). *, *p*<0.05; ***, *p*<0.001. Average values of three independent experiments are shown. **(C)** Immunoblot and immunoprecipitation employing lung tissues were performed (n=4). Representative blots of three independent experiments are shown. **(D)** Immunohistochemical staining was performed. Representative images of the staining are shown (n=3). Quantiﬁcation was performed using Image J (NIH). ***, *p*<0.001.


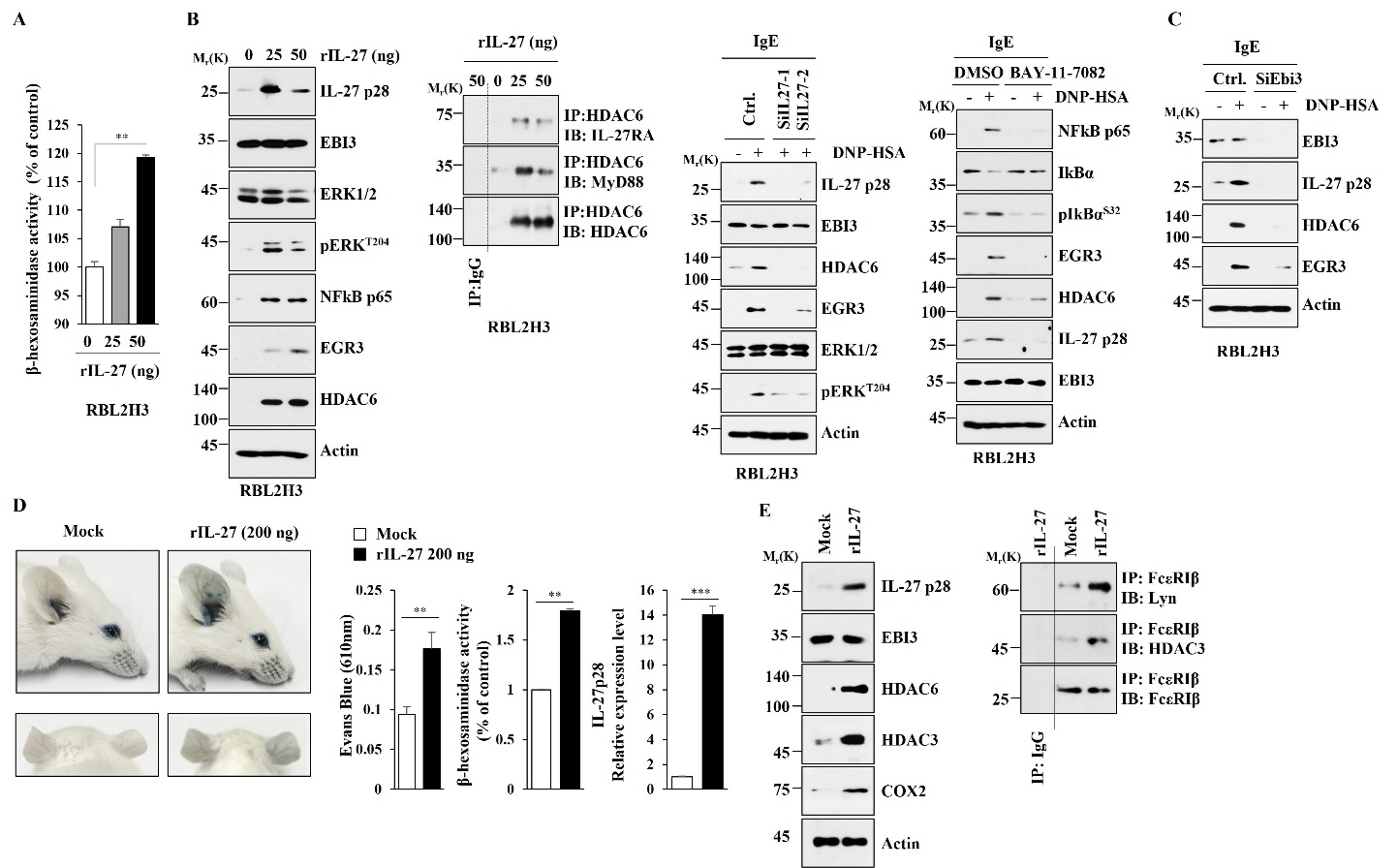


**Figure S6.** Mouse recombinant IL-27 protein induces features of allergic inflammations both *in vitro* and *in vivo*. **(A)** RBL2H3 cells were treated with mouse recombinant IL-27 protein at the indicated amount for 2 h, followed by β-hexosaminidase activity assays. **, *p*<0.01. Average values of three independent experiments are shown. **(B)** RBL2H3 cells were treated with the indicated amount of mouse recombinant IL-27 protein for 2 h, followed by Immunoblot and immunoprecipitation (left). Representative blots of three independent experiments are shown. The indicated siRNA (each at 10 nM) was transfected into RBL2H3 cells. At 24 hours after transfection, cells were sensitized with IgE for 24 h, followed by stimulation with DNP-HSA for 1 h (middle). Representative blots of three independent experiments are shown. The IgE-sensitized RBL2H3 cells were treated without or with BAY-11-7082 (10 μM) for 1 h. Cells were then stimulated without or with DNP-HSA for 1h, followed by immunoblot (right). Representative blots of three independent experiments are shown. **(C)** The indicated siRNA (each at 10 nM) was transfected into RBL2H3 cells. At 24 hours after transfection, cells were sensitized with IgE for 24 h, followed by stimulation with DNP-HSA for 1 h. Representative blots of three independent experiments are shown. **(D)** BALB/C mice were subjected to an intradermal injection of mouse recombinant IL-27 protein (200 ng) for 2 h. Each experimental group comprised five mice (n=5). Vascular permeability was determined as described (n=5). Ear tissue lysates were subjected to qRT-PCR (n=3) and β-hexosaminidase activity assays (n=3). **, *p*<0.01; ***, *p*<0.001. Average values of three independent experiments are shown. **(E)** Immunoblot and immunoprecipitation of ear tissue lysates were performed (n=3). Representative blots of three independent experiments are shown.


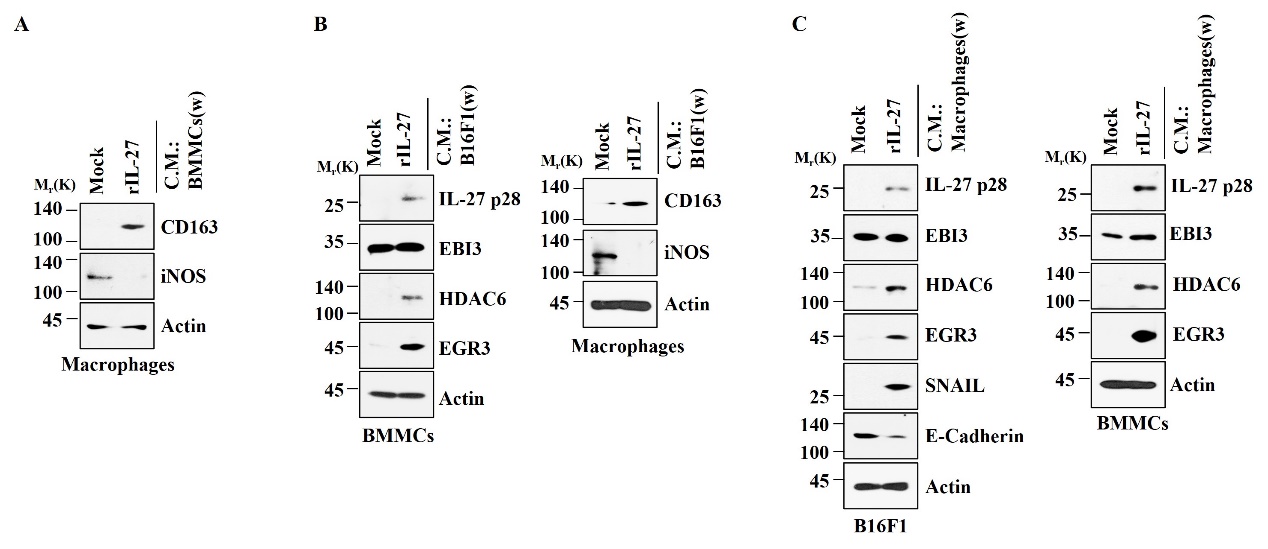


**Figure S7.** Mouse recombinant IL-27 protein promotes cellular interaction involving cancer cells, mast cells, and lung macrophages. **(A)** Culture medium of BMMCs treated with mouse recombinant IL-27 protein (20 ng/ml) for 2 h was removed and replaced with serum-free DMEM. At 12 h after, culture medium was added to lung macrophages for 16 h. Representative blots of three independent experiments are shown. (w) denotes removal of culture medium of the cells treated with mouse recombinant IL-27 protein. **(B)** Culture medium of B16F1 cells treated with mouse recombinant IL-27 protein (20 ng/ml) for 2 h was removed and replaced with serum-free DMEM. At 12 h after, culture medium was added to BMMCs or lung macrophages for 16 h. Representative blots of three independent experiments are shown. (w) denotes removal of culture medium of the cells treated with mouse recombinant IL-27 protein. **(C)** Culture medium of lung macrophages treated with IL-27 protein (20 ng/ml) for 2 h was removed and replaced with serum-free DMEM. At 12 h after, culture medium was added to B16F1 cells or BMMCs for 16 h. Representative blots of three independent experiments are shown. (w) denotes removal of culture medium of the cells treated with mouse recombinant IL-27 protein.


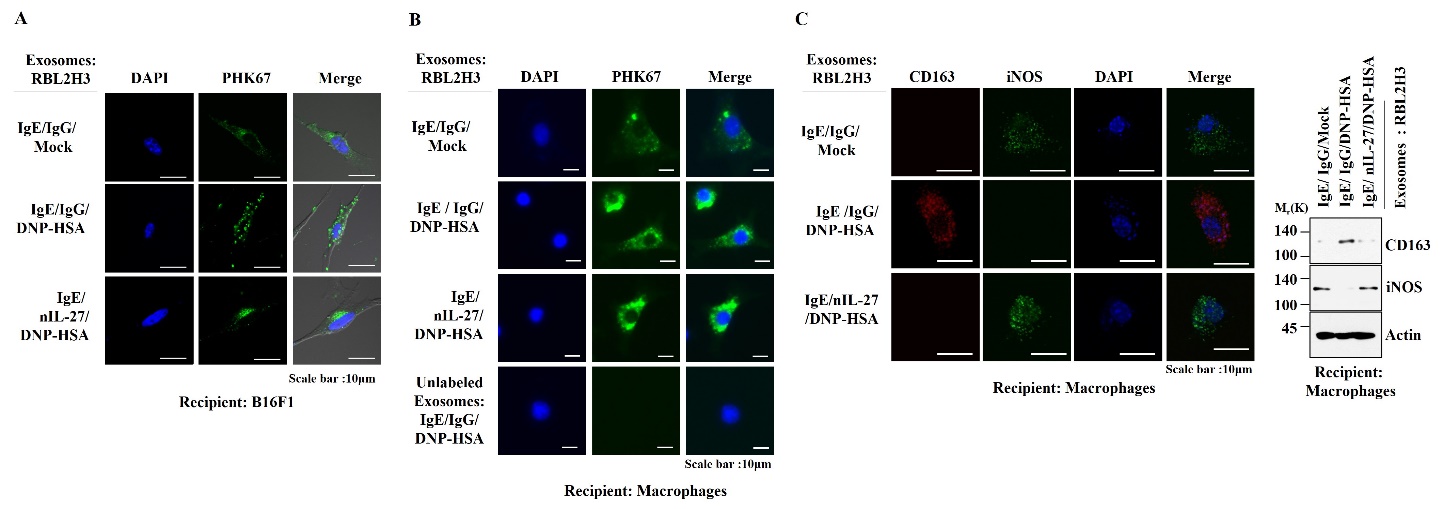


**Figure S8.** Exosomes shuttle between cells. **(A)** PKH67-labeled exosomes (2 μg) from RBL2H3 cells treated, as indicated, were added to B16F1 cells for 24 h. Cells were then visualized using EVOS FL Auto 2 (ThermoFisher, USA). **(B)** Unlabeled or PKH67-labeled exosomes (2 μg) from RBL2H3 cells treated as indicated were added to lung macrophages for 24 h. **(C)** Immunofluorescence staining and immunoblot were performed in lung macrophages. Representative blots of three independent experiments are shown.


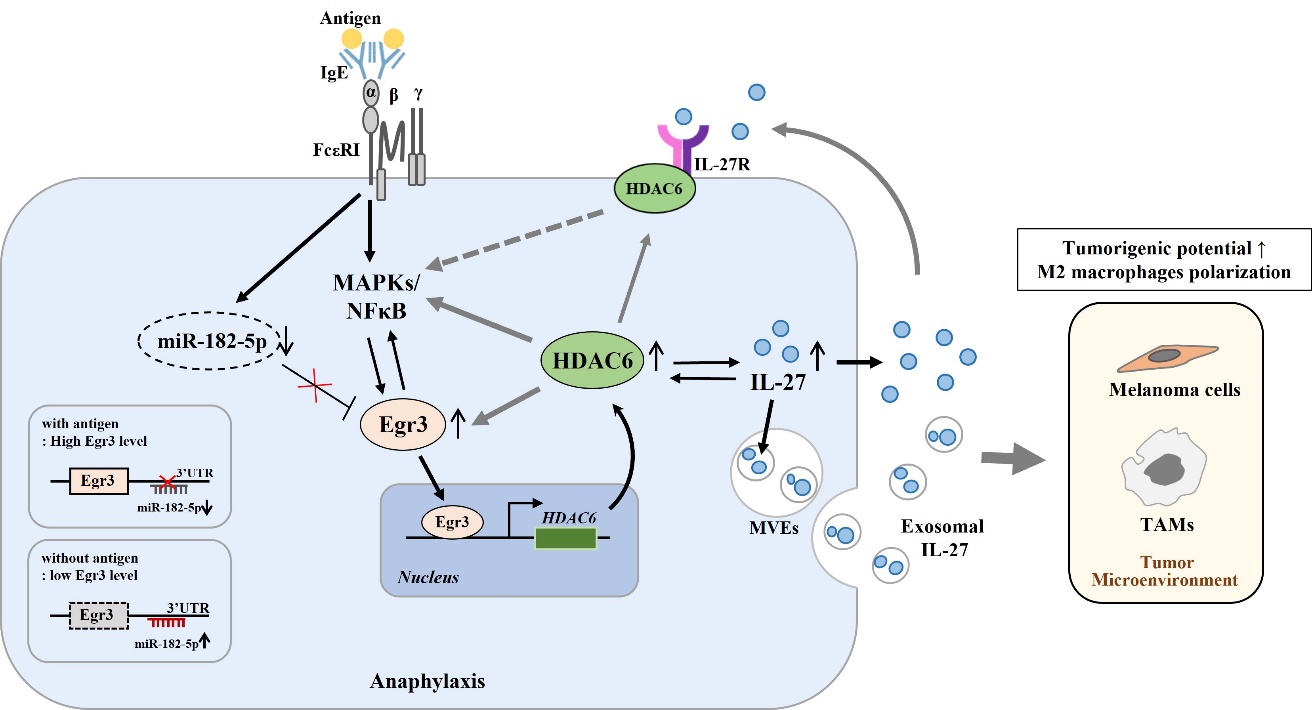


**Figure S9.** The role of EGR3-HDAC6-IL-27 axis in cellular interactions that are necessary for allergic inflammation and tumorigenic and metastatic potentials enhanced by allergic inflammation.

Antigen stimulation increases Egr3 expression via MAPK and NF-kB p65. EGR3 binds to the promoter sequences of HDAC6 to increase HDAC6 expression. miR-182-5p binds to the 3' UTR of Egr3 to decrease Egr3 expression. HDAC6 is necessary for the increased expression levels of pERK/NF-kB p65 and EGR3 by antigen stimulation. HDAC6 increases IL-27 expression and binds to IL-27 receptor. IL-27 may amplify FcεRI signaling involving NF-kB p65 and HDAC6. IL-27 is present in exosomes and mediate cellular interactions involving cancer cells, mast cells, and macrophages which are necessary for tumorigenic potential enhanced by allergic inflammation.
