# Supplementary material for: EGR3-HDAC6-IL-27 Axis Mediates Allergic Inflammation and Is Necessary for Tumorigenic Potential of Cancer Cells Enhanced by Allergic Inflammation-Promoted Cellular Interactions
Source: Front Immunol. 2021 Jun 21;12:680441. doi: 10.3389/fimmu.2021.680441 (PMC8257050; doi:10.3389/fimmu.2021.680441)
Supplement: Supplementary file 2 [file DataSheet_2.docx]

**Supplementary Table 1.** The sequences of microRNA mimics

| **Name** | **Company** | **Sequence (5'-3')** |
| --- | --- | --- |
| Negative control | Dharmacon | miRIDIAN microRNA Mimic Negative Control #1 (Cat.CN-001000-01-05) |
| miR-182-5p | Dharmacon | UUUGGCAAUGGUAGAACUCACACCG |

**Supplementary Table 2.** The sequences of SiRNAs

| **Name** | **Company** | **Sequence (5'-3')** |
| --- | --- | --- |
| Negative control | Bioneer | AccuTarget™ Negative Control siRNA (Cat. SN-1002) |
| SiEGR3  (Rat) | Bioneer | sense: CAGAGAAUGUGAUGGACAU |
|  |  | antisense: AUGUCCAUCACAUUCUCUG |
| SiEGR3  (Mouse) | Bioneer | sense: CAGAUAGAAGUGAAGUGUA |
|  |  | antisense: UACACUUCACUUCUAUCUG |
| SiHDAC6  (Rat) | Sigma Aldrich | SASI_Rn02_00288015 (Cat. PDSIRNA5D) |
| SiHDAC6  (Mouse) | Bioneer | sense: ACUAGACAGCGAAAGAGUATT |
|  |  | antisense: UACUCUUUCGCUGUCUAGUTT |
| SiIL27 p28 -1  (Mouse) | Bioneer | sense: GCUACCCUUGCUUCUGGUA |
|  |  | antisense: UACCAGAAGCAAGGGUAGC |
| SiIL27 p28 -2  (Mouse) | Bioneer | sense: GCUUCUGGUACAAGCUGGU |
|  |  | antisense: ACCAGCUUGUACCAGAAGC |
| SiEBI3  (Rat) | Bioneer | sense: GUCACUAACUCGGAUCCAA |
|  |  | antisense: UUGGAUCCGAGUUAGUGAC |

**Supplementary Table 3.** Primer sequences for qRT-PCR

| **Name** | **Sequence (5'-3')** |
| --- | --- |
| U6 | TGGCCCCTGCGCAAGGATG |
| miR-182b-5p | TTTGGCAATGGTAGAACTCACACCG |
| HDAC6 (Mouse) | F: CCTCAGCGCATCTTACGCAT |
|  | R: CAGCACTGTGGCAGGTAAGG |
| HDAC6 (Rat) | F: TCATCAGGCCTCCTGGACAC |
|  | R: TGACCATGGTGCACATCCCA |
| EGR3 (Mouse) | F: GACAATCTGTACCCCGAGGAG |
|  | R: TCCATCACATTCTCTGTAGCCATC |
| EGR3 (Rat) | F: TGCCTGACAATCTGTACCCC |
|  | R: TCCATCACATTCTCTGTAGCCA |
| IL27 p28 (Mouse) | F: TTTCCAGGCATGGCATCACC |
|  | R: CACAGCTGCTCCCTCTCTGA |
| IL27 p28 (Rat) | F: AATTGGGTCTGGAGGTGAGGA |
|  | R: GTTCTGTTAGTGAGGGCCAGT |
| Actin (Mouse) | F: ATGTGGATCAGCAAGCAGGA |
|  | R: CTAGAAGCACTTGCGGTGC |
| Actin (Rat) | F: ACCCGCGAGTACAACCTTCT |
|  | R: TCGTCATCCATGGCGAACT |
| EBI3 (Mouse) | F: CAAGTACCGACTCCGCTACC |
|  | R: GGGTTTCGAGTTCCTGAGGG |
| EBI3 (Rat) | F: CCACAGTGCCCTACTTGCTA |
|  | R: GATTCGTTCCGCCACAAAGG |

**Supplementary Table 4.** Primary antibodies used for immunoblot, immunofluorescence, ChIP assay, neutralization, immunogold labeling and immunohistochemical staining

| Catalog No. | Company | Name |
| --- | --- | --- |
| A11259 | Abclonal | HDAC6 |
| A11225 |  | TLR2 |
| A5258 |  | TLR4 |
| sc-393789 | Santa Cruz | FcεRIβ |
| sc-7274 |  | Lyn |
| sc-1648 |  | JNK1 |
| sc-6254 |  | pJNK1^T183/Y185^ |
| sc-74532 |  | MyD88 |
| sc-7964 |  | TSG101 |
| sc-166029 |  | CD81 |
| sc-518028 |  | SOCS1 |
| sc-390967 |  | EGR3 |
| sc-271977 |  | SNAI1 |
| sc-166158 |  | EBI3 |
| #4695 | Cell signaling | ERK1/2 |
| #4370 |  | pERK^T204^ |
| #8690 |  | p38MAPK |
| #4511 |  | p-p38MAPK^T180/Y182^ |
| #8242 |  | NFκB p65 |
| #4812 |  | IKBα |
| #2859 |  | pIKBα^S32^ |
| #3949 |  | HDAC3 |
| #12282 |  | COX2 |
| #3195 |  | E-Cadherin |
| #13120 |  | iNOS |
| 66009-1-Ig | Proteintech | Beta-actin |
| ab182422 | Abcam | CD163 |
| ab115819 |  | iNOS |
| AF1834 | R&D | IL27 p28 |
| AHP1620 | BIO-RAD | IL27R alpha |

**Supplementary Method**

**β-hexosaminidase Activity Assays**

RBL2H3 cells were grown on 2% (w/v) BSA-coated 96 well plates (2×10^5^ cells/well) and sensitized for 16 h with DNP-specific IgE (100 ng/ml). The culture medium was replaced with Tyrodes' assay buffer (119 mM NaCl, 4.74 mM KCl, 2.5 mM CaCl2, 1.19 mM MgSO4, 10 mM 4-2-hydroxyethyl-1-piperazineethane sulfonic acid (HEPES), 5 mM glucose, 0.1% (w/v) BSA, pH7.3). IgE-sensitized cells were then preincubated with the above buffer for 15 min. The cells were then stimulated with DNP-HSA (100 ng/ml) for 1 h. The supernatant (80 μl) was incubated with an equal volume of substrate solution (1 mM p-nitrophenyl N-acetyl-beta-D-glucosamine in 0.05 M citrate buffer, pH 4.5) for 1 h at 37 °C. The enzyme reaction was stopped by the addition of 0.05 M sodium bicarbonate buffer (pH 10.0) and the reaction product was measured at 450 nm. To determine the total amount of β-hexosaminidase released, the remaining cells were lysed by assay buffer containing 1% (v/v) Triton X-100 prior to incubation with substrate. For β-hexosaminidase assay using tissue lysates, tissue was frozen in liquid nitrogen to preserve protein structure and homogenized using lysis buffer (62.5 mm Tris-HCl, pH 6.8, 2% (w/v) SDS, 10% (v/v) glycerol, 50 mm dithiothreitol, 0.01% (w/v) bromophenol blue, 10 mm NaF, 1% (v/v) protease inhibitor mixture (Roche), 1 mm sodium orthovanadate). Vortexing and centrifugation at 10,000 X g for 15 min at 4^o^C were followed. Supernatant was then obtained and used as tissue lysates. Eighty μl of tissue lysates (50 μg/ml) were incubated with an equal volume of substrate solution for 1 h at 37 °C. The enzyme reaction was stopped by the addition of 0.05 M sodium bicarbonate buffer (pH 10.0) and the reaction product was measured at 450 nm.

**Immunoblot and Immunoprecipitation**

Cells were solubilized by lysis buffer (62.5 mm Tris-HCl, pH 6.8, 2% (w/v) SDS, 10% (v/v) glycerol, 50 mm dithiothreitol, 0.01% (w/v) bromophenol blue, 10 mm NaF, 1% (v/v) protease inhibitor mixture (Roche), 1 mm sodium orthovanadate). For immunoblot, lysates were mixed with 2 X sample buffer, boiled for 5 min, and denatured proteins (10-20 μg) were analyzed on 10% SDS-PAGE. After electrophoresis, proteins were transferred to PVDF membrane. After transfer, membrane was incubated with blocking solution (5% BSA in TBS-T buffer) for 30 minutes to block non-specific binding. The membrane was then incubated with primary antibody (diluted in 2% BSA) at 4°C for overnight, followed by washing three times with TBS-T for 15 min and incubation with anti-mouse or anti-rabbit horseradish peroxidase-conjugated antibody for 1 h. Immunodetection was performed using an enhanced chemiluminescent substrate solution (GenDEPOT, USA).

To isolate tissue lysates, tissue was frozen in liquid nitrogen to preserve protein structure and homogenized using lysis buffer. Vortexing and centrifugation at 10,000 X g for 15 min at 4^o^C were followed. Supernatant was then obtained and used as tissue lysates.

For immunoprecipitations, cell lysates or tissue lysates (100-200 μg) were immunoprecipitated with respective primary antibody (0.2 -2 μg) for overnight at 4℃. Twenty μl of Protein A/G PLUS-Agarose (Santa Cruz) was then added and incubation was continued for 1 hour at 4℃. Beads were washed three times with lysis buffer, 2X sample buffer was added. Samples were then denatured (100 °C for 5min) and analyzed 10% SDS-PAGE, followed by immunoblot.
